# Synthesis and Structural Characterization of Novel Dimers of Dipyridothiazine as Promising Antiproliferative Agents

**DOI:** 10.3390/molecules28227662

**Published:** 2023-11-19

**Authors:** Emilia Martula, Beata Morak-Młodawska, Małgorzata Jeleń, Patrick N. Okechukwu, Abbirami Balachandran, Prethika Tehirunavukarasu, Kirthani Anamalay, Vaidehi Ulaganathan

**Affiliations:** 1 Doctoral School of The Medical University of Silesia, 40-055 Katowice, Poland; d201074@365.sum.edu.pl; 2 Department of Organic Chemistry, Faculty of Pharmaceutical Sciences, The Medical University of Silesia, Jagiellońska 4, 41-200 Sosnowiec, Poland; manowak@sum.edu.pl; 3 Department of Biotechnology, Faculty of Applied Sciences, UCSI University, Cheras, Kuala Lumpur 56000, Malaysia; patrickn@ucsiuniversity.edu.my (P.N.O.); 1001233455@ucsiuniversity.edu.my (A.B.); prethika18@gmail.com (P.T.); kirthani12@hotmail.com (K.A.); 4 Department of Food Science and Nutrition, Faculty of Applied Sciences, UCSI University, Cheras, Kuala Lumpur 56000, Malaysia; vaidehi@ucsiuniversity.edu.my

**Keywords:** phenothiazines, dimers of dipyridothiazines, structural analysis, anticancer action, selectivity index, cytotoxicity

## Abstract

Many new isomeric dipyridothiazine dimers have been presented as molecules with anticancer potential. These compounds were obtained in efficient syntheses of 1,6-, 1,8-, 2,7- and 3,6-diazaphenothiazines with selected alkylaromatic linkers. The structures of these compounds has been proven with two-dimensional spectroscopic techniques (COSY, NOESY, HSQC and HMBC) and high-resolution mass spectrometry (HRMS). In silico analyses of probable molecular targets were performed using the Way2Drug server. All new dimers were tested for anticancer activity against breast cancer line MCF7 and colon cancer line SW480. Cytotoxicity was assessed on normal L6 muscle cells. The tested dimers had high anticancer potential expressed as IC_50_ and the selectivity index SI. The most active derivative, **4c**, showed an IC_50_ activity of less than 1 µM and an SI selectivity index higher than 100. Moreover, the compounds were characterized by low toxicity towards normal cells, simultaneously indicating a high cytostatic potential.

## 1. Introduction

Despite innovative therapeutic approaches being constantly discovered, cancer remains one of the main causes of human mortality around the world [1]. According to the latest reports, we lose about 10 million people to cancer every year, with the most common cases among women being breast cancer and among men being prostate and colon cancer. This fact indicates the need for further research to be undertaken in anticancer field therapies [1,2,3]. On the one hand, there is a return to existing therapeutics and research is being carried out towards their different activity; on the other hand, ongoing analyses are being carried out to design and synthesize new molecules whose activity is focused on anticancer potential [4].

Heterocyclic systems play an essential role in medicinal chemistry and are related to the search for valuable new drug candidates [5,6]. One of the most biologically active cores is a six-membered pyridine ring, which is a building block found in many medicinal substances, such as sorafenib, zapiclone, isoniazid, tazarotene and bromazepam [7,8,9,10,11]. At the same time, it should be remembered that its derivatives also include nicotine, vitamin PP and the nucleotides NADH and NAD+ [12,13,14].

Other pharmacologically important heterocyclic derivatives are dibenzo-1,4-thiazines (chlorpromazine, thioridazine or fluphenazine), which revolutionized psychiatry in the 1950s by containing valuable neuroleptic and antipsychotic properties [15]. Research in this group of drugs is ongoing and shows other valuable properties of these molecules, such as anticancer, antibacterial, antioxidant and reversing multidrug resistance [16,17,18,19]. Modified derivatives of phenothiazines are dipyridothiazines [20], whose structure contains two pyridine rings instead of benzene rings. As proven in the literature, these compounds have high anticancer, immunomodulatory and antioxidant potential [21,22,23,24].

The most promising dipyridothiazines showed cytotoxic activity of less than 1 μM and a relatively low cytotoxicity to normal human cells. Gene expression analysis connected with *H3 * (*gene encoding proliferation marker histone H3*), *TP53* (*tumor suppressor gene*), *CDKN1A* (*gene of cell cycle inhibitor*), *BCL-2* (*antiapoptotic gen*) and *BAX* (*proapoptotic gen*) performed with RT-QPCR (*real-time quantitative reverse transcription PCR*) indicated the possibility of inducing apoptosis through the mitochondrial pathway [21].

Considering the biological potential of dipyridothiazines, we decided to synthesize new dipyridothiazine dimers because numerous reports in the literature showed an enhancement of the biological activity of bisheterocycles containing an appropriate spacer above the heterocyclic chemical system [25,26]. Additionally, there are only two reports in the literature showing bis-phenothiazine systems that exhibited valuable antibacterial and antituberculosis activities [27,28]. We rationally designed the synthesis of new dipyridothiazine dimers, combining bioactive dipyridothiazines (1,6-, 1,8-, 2,7- and 3,6-diazaphenothiazine **1**–**4**) with selected alkylaromatic linkers into dimers systems (**1a,b,c**–**4a,b,c**) using the alkylation reaction. The structures were clearly proven with modern spectroscopic techniques (NMR, 2D NMR (*two-dimensional NMR*) and HRMS). For new derivatives, in silico analyses of probable molecular targets were performed using the Way2Drug server. Anticancer activity was appointed on– selected cancer cell lines: MCF7 breast cancer and SW480 colon cancer. Cytotoxicity was determined against normal L6 myoblast cells isolated from rat skeletal muscle. The conducted research shows the anticancer potential of new dimer systems containing selected dipyridothiazines in their structure.

## 2. Results and Discussion

### 2.1. Chemistry Part

**Scheme 1:**
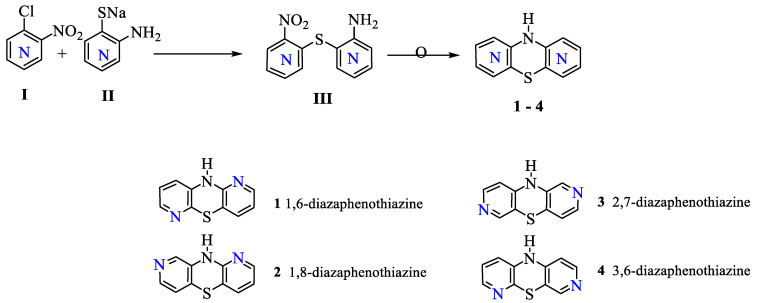
Paths for the synthesis of building blocks **1**–**4** that are substrates of the final dimers [29,30,31,32].

The synthetic strategy used to prepare new dipyridothiazine dimers was based on the selection of previously obtained diazaphenothiazines **1**–**4**, which are fundamental. The starting substances, comprising 10*H*-1,6-, 1,8-, 2,7- and 3,6-diazaphenothiazines **1**–**4**, were obtained using multi-stage syntheses with substrates, which were, respectively, disubstituted pyridine derivatives **A** and **B**. These compounds allowed us to selectively obtain isomeric sulfides **C**, which were subjected to reactions in accordance with the literature data through the Smiles rearrangement and led to the formation of four isomeric dipyridothiazines **1**–**4** (Scheme 1) [29,30,31,32].

In the next step, selected dipyridothiazines were subjected to alkylation reactions with selected linkers (α,α′-dichloro-*o*-xylene **a**, α,α′-dichloro-*p*-xylene **b** and 2,6-bis(chloromethyl)pyridine **c**).

**Scheme 2:**
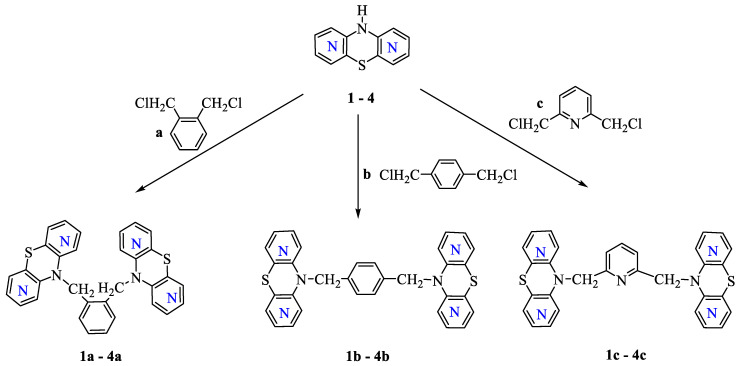
Synthesis of final dimer derivatives of dipyridothiazines **1a,b,c**–**4a,b,c**.

The reactions were carried out in a dry DMF in the presence of the strong base NaH, which led to the final dimer systems **1a,b,c**–**4a,b,c** (Scheme 2). The crude products of the reactions were separated with column chromatography to obtain pure final derivatives. All new compounds were obtained with good yields (79–88%).

It is worth noting that in each of the obtained dimers, two dipyridothiazine units were linked to labile alkylaromatic linkers, which did not stiffen the structure of the molecules.

### 2.2. Structural ^1^H, ^13^C NMR and HRMS Study

The identification of the molecule structure was based on ^1^H, ^13^C NMR spectra and 2D NMR experiments: COSY (*Correlation Spectroscopy*), NOESY (*Nuclear Overhauser Effect Spectroscopy*) and mass spectrometry HRMS (*High-Resolution Mass Spectrometry*). Derivatives **1a,b**–**4a,b** are compounds with an axis of symmetry in their structure passing through the benzene ring in the liner, which is why only half of the protons of these molecules were observed in the ^1^H NMR spectra. However, derivatives **1c**–**4c** containing the lutidine system were structurally presented in ^1^H NMR as whole molecules.

**Figure 1:**
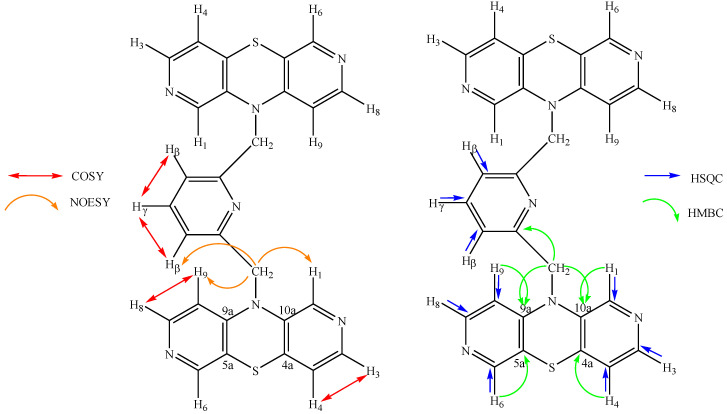
The ^1^H and ^13^C NMR assignment and ^1^H-^1^H (COSY and NOESY), ^1^H-^13^C (HSQC and HMBC) connections in compound **3c**.

Considering modern standards of structural analysis, the structure of the obtained products had to be clearly confirmed. For this reason, a two-dimensional experiment was carried out, allowing the assignment of individual places to protons and carbons in the spectra. For this purpose, the dimeric derivative **3c** was used, and the results of the interactions of individual protons and carbons are summarized in Table 1 and illustrated in Figure 1. The spectra are included in the Supplemental Materials Section. 

The **3c** dimer is composed of two 2,7-diazaphenothiazine units, which are connected by labile CH_2_ groups to the substituent pyridine system in positions 2 and 6. Therefore, in the ^1^H spectrum, a double number of six proton signals of the 2,7-diazaphenothiazine system, a double proton β signal pyridine and a characteristic single γ proton signal is observed. The problem of how to distinguish which proton signals come from a specific hydrogen atom was resolved using the NOESY spectrum. This spectrum showed the interaction of the singlet at 4.99 ppm and corresponds to the CH_2_ group with the singlet at 7.48 ppm (which was assigned to the proton H_1_) as well as the interaction with the doublet at 6.02 ppm (which was assigned to the proton H_9_ and the doublet at 7.34 ppm, which was assigned to the β proton of pyridine). Then, the COSY correlation spectrum was analyzed, which shows the interactions of the H_9_ signal with H_8_ and H_β_ with H_γ_ as well as the H_4_ signals with H_3_, which were assigned accordingly. Individual protons were assigned in this way (Figure 1).

In a further step, which used the HSQC (*Heteronuclear Single Quantum Correlation*) spectrum, the carbon atoms adjacent to the protons were assigned appropriately. However, the interactions of these protons through two and three bonds observed in the HMBC (*Heteronuclear Multiple Bond Correlation*) spectrum allowed chemical shifts to be assigned to quaternary carbons (Table 1). High-resolution mass spectrometry analysis confirmed the molecular mass of the tested dimer **3c** and its purity.

### 2.3. In Silico Target Prediction

For all new compounds, preliminary in silico analyses of probable molecular targets were performed using the Way2Drug server, which includes the PASS program (*prediction of activity spectra for substances*) and allows to predict probable biological activities and mechanisms of action [33]. The probability indicators of biological activity, Pa, expressed in %, are included in Table 2.

The PASS program indicated that the studied group of dimers has a high potential for probable anticancer activities, which are related to the stimulation mechanism of histone deacetylase, inhibition of the p21 protein and the mitochondrial apoptosis pathway. It is worth noting that the probability of activity in neurodegenerative diseases as well as the antioxidant activity related to the mechanism of cytochrome 450 stimulation have been indicated.

### 2.4. Biology Part

Previous promising results regarding the antiproliferative effect of dipyridothiazines [29,30,31,32], as well as the results obtained from in silico predictions, prompted us to investigate the biological potential of new dipyridothiazine dimers. The antiproliferative activity of all 12 new derivatives was tested in vitro against cancer cell lines: MCF 7 breast cancer and SW480 colon cancer using the MTT assay. A normal L6 rat myoblast was used as a control. The obtained results are presented in Table 3.

Additionally, the SI (selectivity index) was calculated for each compound using the following formula: SI = (IC_50_ for normal cell line **L6**)/(IC_50_ for respective cancerous cell line). The selectivity index results are included in Table 4.

Doxorubicin was used as a positive control to induce cell death. The results of the cytotoxicity studies are summarized in Table 3. The obtained results showed that the new **1a,b,c**–**4a,b,c** dimers were not toxic to the normal L6 cell line in the range of tested concentrations both after 24, 48 and 72 h of incubation. Dimers **1c**, **2a**, **2b**, **3a** and **4c** were significantly toxic towards both MCF7 and SW480 cancer cells, while derivatives **1a**, **1b** and **2c** showed a higher cytotoxicity and therefore a certain selectivity towards the breast cancer cell line MCF7 than the colon cancer cell line SW480.

In the group of dimers constructed using 1,6-diazaphenothiazine, the highest cytotoxicity towards both cancer cell lines was demonstrated by derivative **1c** containing lutidine in its structure (IC_50_ in the range of 0.67–5.0 µM). This activity is three times higher than that of doxorubicin. However, derivatives **1a** and **1b** showed selectivity towards breast cancer cells (Table 4.). In the group of dimers containing 1,8-diazaqphenothiazine in their structure, derivative **2a** had the highest cytotoxicity containing o-xylene in its structure (IC_50_ in the range of 6.27–9.68 µM). The remaining derivatives of this group had activity comparable to the reference drug. A similar situation was observed in the group of derivatives with 2,7-diazaphenothiazine, where derivative **3a** was also highly active (IC_50_ in the range of 3.17–6.12 µM). Moreover, the group of these dimers is characterized by a positive selectivity index (Table 4.). When analyzing the results in the group of dimers containing 3,6-diazaphenothiazine, derivative **4c**, containing lutidine in its structure, attracts attention. This compound showed the highest activity towards MCF 7 cancer cells. The analysis of the IC_50_ value showed nanomolar activity towards breast cancer cells in the range of the tested concentrations (IC_50_ was 0.12 µM after 24 h; IC_50_ was 2.112 × 10^−5^ µM after 48 h; IC_50_ value was outside the tested concentration range after 72 h). This activity was many times higher than the activity of the reference drug (Table 3). The selectivity index (SI) of this dimer is higher than 100. This derivative has the highest anticancer potential in this group of dimers.

The tested dimers (**1a,b,c**–**4a,b,c**) were characterized by significant antiproliferative activity in relation to the tested cancer cell lines and relatively weak cytotoxicity in relation to normal cells. This activity largely depended on the type of isomeric dipyridothiazine constituting the dimer system and on the linker connecting two thiazine units. The obtained results are promising and show the anticancer potential of dipyridothiazine dimers.

## 3. Materials and Methods

### 3.1. Chemistry

Melting points were determined in open capillary tubes on a Boetius melting point apparatus and were uncorrected. The standard NMR spectra were recorded on Bruker Avance spectrometers (^1^H at 600 MHz, ^13^C at 150 MHz) in CDCl_3_ and DMSO_d6_. Two-dimensional COSY, NOESY, HSQC and HMBC spectra of selected compounds were recorded on a Bruker Avance spectrometer at 600 MHz, using COSYGPSW, NOESYGPPHSW, HSQCGPPH and HMBCGP experiments. The HRMS spectra (EI—electro impact ionization) were run on a Brucker Impact II. Thin-layer chromatography was performed on silica gel 60 F_254_ (Merck 1.05735) with CHCl_3_-EtOH (10:1 *v*/*v*) and on aluminum oxide 60 F_254_ neutral (type E) (Merck 1.05581) with CHCl_3_-EtOH (10:1 *v*/*v*) as eluents.

10*H*-1,6-Diazaphenothiazine (**1**), 10*H*-1,8-diazaphenothiazine (**2**), 10*H*-2,7-diazaphenothiazine (**3**) and 10*H*-3,6-diazaphenothiazine (**4**) were obtained and purified according to previously described methods (Scheme 1) [29,30,31,32].

#### 3.1.1. General Procedure for Synthesis of Compounds (**1a,b,c**–**4a,b,c**)

To a solution of selected diazaphenothiazine (**1**–**4**) (0.201 mg, 1 mmol) in dry DMF (10 mL), NaH (0.028 g, 1.2 mmol, 60% NaH in mineral oil washed out with hexane) was added. The reaction mixture was stirred at room temperature for 1 h; then, selected linkers (α,α′-dichloro-o-xylene **a**, α,α′-dichloro-p-xylene **b** and 2,6-bis(chloromethyl)pyridine **c**), 0.5 mmol) were added and stirring was continued for 24 h. The mixture was poured into water (15 mL), extracted with CHCl_3_ (3 × 10 mL) and dried using Na_2_SO_4_. The obtained product was purified with column chromatography (aluminum oxide, CHCl_3_-Ethanol 10:1) to provide the following information about the dimers: 

##### Dimers of 1,6-Diazaphenothiazines **1a,b,c**

1,2-bis((dipyrido[2,3-b:2′,3′-e][1,4]thiazin-10-yl)methyl)benzene (**1a**) (yield 83%), mp 265–267 °C.

^1^H NMR (CDCl_3_) δ: 5.35 (s, 2H, CH_2_), 6.79 (m, 2H), 6.86 (m, 1H), 7.16 (m, 1H), 7.25 (s, 2H_ph_), 7.27 (s, 1H), 7.92 (dd, 1H), 7.95 (dd, 1H). ^13^C NMR: 46.40, 116.51, 118.58, 121.64, 121.97, 127.13, 127.60, 133.44, 134.56, 138.40, 142.87, 144.67, 145.35, 152.35. HRMS (EI) *m*/*z* for [C_28_H_21_N_6_S_2_ + H], calc. 505.1264, found: 505.1274.

1,4-bis((dipyrido[2,3-b:2′,3′-e][1,4]thiazin-10-yl)methyl)benzene (**1b**) (yield 85%), mp 160–161 °C.

^1^H NMR (CDCl_3_) δ: 5.54 (s, 2H, CH2), 6.70 (dd, 1H), 6.78 (m, 2H), 7.24 (s, 2Hph), 7.26 (s, 1H), 7.91 (m, 2H). ^13^C NMR: 116.22, 118.50, 121.34, 121.85, 126.87, 127.50, 134.50, 135.45, 138.38, 142.74, 144.35, 145.30, 152.37. HRMS (EI) *m*/*z* for [C_28_H_21_N_6_S_2_ + H], calc. 505.1264, found: 505.1260.

2,6-bis((dipyrido[2,3-b:2′,3′-e][1,4]thiazin-10-yl)methyl)pyridine (**1c**) (yield 80%), mp 245–247 °C.

^1^H NMR (CDCl_3_) δ: 5.28 (s, 4H, 2 CH_2_), 6.69 (d, 2H), 6.74 (m, 4H), 7.15 (m, 2H_βpyr_), 7.23 (d, 2H), 7.54 (t, 1H_γpyr_), 7.79 (dd, 2H), 7.91 (d, 3H). ^13^C NMR: 50.50, 115.94, 118.40, 119.60, 121.20, 121.75, 134.22, 137.34, 138.51, 142.59, 144.05, 144.97, 152.05, 156.76. HRMS (EI) *m*/*z* for [C_27_H_20_N_7_S_2_ + H], calc. 506.1216, found: 506.1223.

##### Dimers of 1,8-Diazaphenothiazines **2a,b,c**

1,2-bis((dipyrido[3,2-b:3′,4′-e][1,4]thiazin-10-yl)methyl)benzene (**2a**) (yield 82%), mp 260–261 °C.

^1^H NMR (CDCl_3_) δ: 5.43 (s, 2H, CH_2_), 6.80 (m, 1H), 6.97 (d, 1H), 7.15 (m, 1H), 7.20 (m, 1H), 7.25 (m, 1H), 7.91 (s, 1H), 8.01 (m, 2H). ^13^C NMR: 46.20, 115.02, 118.81, 120.87, 125.68, 127.36, 133.15, 134.61, 135.56, 138.80, 143.18, 146.23, 153.01, 154.91. HRMS (EI) *m*/*z* for [C_28_H_21_N_6_S_2_ + H], calc. 505.1264, found: 505.1289.

1,4-bis((dipyrido[2,3-b:2′,3′-e][1,4]thiazin-10-yl)methyl)benzene (**2b**) (yield 86%), mp 184–186 °C.

^1^H NMR (CDCl_3_) δ: 5.30 (s, 2H, CH_2_), 6.79 (m, 1H), 6.91 (d, 1H), 7.20 (dd, 1H), 7.21 (s, 2H_ph_), 7.22 (s, 2H_ph_), 7.77 (s, 1H), 7.96 (dd, 1H), 7.98 (d, 1H). ^13^C NMR: 48.19, 114.13, 118.70, 120.70, 127.01, 134.37, 134.95, 135.19, 138.63, 142.77, 145.97, 153.09. HRMS (EI) *m*/*z* for [C_28_H_21_N_6_S_2_ + H], calc. 505.1264, found: 505.1291.

2,6-bis((dipyrido[3,2-b:3′,4′-e][1,4]thiazin-10-yl)methyl)pyridine (**2c**) (yield 79%), mp 266–268 °C.

^1^H NMR (CDCl_3_) δ: 5.29 (s, 4H, 2CH_2_), 6.75 (m, 2H), 6.89 (d, 2H), 7.10 (dd, 2H), 7.18 (d, 2H), 7.59 (s, 2Hβ), 7.60 (t, 1Hγ), 7.81 (dd, 2H), 7.90 (d, 2H). ^13^C NMR: 49,89, 112.99, 118.91, 119.86, 120.63, 132.56, 133.91, 137.60, 139.42, 140.56, 146.11, 151.94, 155.56, 162.54. HRMS (EI) *m*/*z* for [C_27_H_20_N_7_S_2_ + H], calc. 506.1216, found: 506.1248.

##### Dimers of 2,7-Diazaphenothiazines **3a,b,c**

1,2-bis((dipyrido[3,4-b:3′,4′-e][1,4]thiazin-10-yl)methyl)benzene (**3a**) (yield 88%), mp 280–282 °C.

^1^H NMR (CDCl_3_) δ: 5.17 (s, 2H, CH_2_), 6.47 (d, 1H), 7.01 (d, 1H), 7.31 (m, 2H), 7.87 (s, 1H), 8.11 (m, 2H), 8.18 (d, 2H). ^13^C NMR: 50.10, 110.06, 117.65, 121.69, 128.61, 129.01, 131.28, 134.04, 136.33, 137.99, 145.02, 146.85, 149.14, 149.63, 150.09. HRMS (EI) *m*/*z* for [C_28_H_21_N_6_S_2_ + H], calc. 505.1264, found: 505.1318.

1,4-bis((dipyrido[2,3-b:2′,3′-e][1,4]thiazin-10-yl)methyl)benzene (**3b**) (yield 86%), mp 215–217 °C.

^1^H NMR (CDCl_3_) δ: 5.07 (s, 2H, CH_2_), 6.45 (d, 1H), 6.98 (d, 1H), 7.26 (s, 2H), 7.82 (s, 1H), 8.09 (m, 2H), 8.15 (d, 1H). ^13^C NMR: 51.49, 109.61, 116.72, 121.42, 127.00, 133.26, 133.91, 135.89, 138.08, 144.84, 146.37, 149.61, 150.14. HRMS (EI) *m*/*z* for [C_28_H_21_N_6_S_2_ + H], calc. 505.1264, found: 505.1296.

2,6-bis((dipyrido[3,4-b:3′,4′-e][1,4]thiazin-10-yl)methyl)pyridine (**3c**) (yield 78%), mp 245–247 °C.

^1^H NMR (CDCl_3_) δ: 4.99 (s, 4H, 2CH_2_), 6.02 (d, 2H, H_9_), 6.96 (d, 2H, H_4_), 7.35 (d, 2H, H_β_), 7.49 (s, 2H, H_1_), 7.51 (d, 2H, H_8_), 7.85 (t, 1H, H_γ_), 7.91 (s, 2H, H_6_), 8.06 (d, 2H, H_3_). ^13^C NMR: 51.89 (2CH_2_), 109.53 C_9_, 115.73 C_4a_, 120.44 C_β_, 121.55 C_4_, 133.16 C_10a_, 135.25 C_1_, 137.82 C_9a_, 138.97 C_γ_, 144.52 C_3_, 145.30 C_6_, 148.38 C_8_, 150.24 C_5a_, 154.90 C_α_. HRMS (EI) *m*/*z* for [C_27_H_20_N_7_S_2_ + H], calc. 506.1216, found: 506.1248.

##### Dimers of 3,6-Diazaphenothiazines **4a,b,c**

1,2-bis((dipyrido[2,3-b:4′,3′-e][1,4]thiazin-5-yl)methyl)benzene (**4a**) (yield 83%), mp 302–303 °C.

^1^H NMR (CDCl_3_) δ: 5.12 (s, 2H, CH_2_), 6.44 (m, 1H), 6.79 (m, 1H), 6.93 (m, 1H), 7.30 (S, 2h), 7.33 (m, 1H), 8.06 (d, 1H), 8.14 (d, 1H), 8.17 (s, 1H).

^13^C NMR: 50.30, 109.81, 119.54, 121.95, 122.08, 128.63, 129.19, 131.50, 137.72, 144.31, 146.24. HRMS (EI) *m*/*z* for [C_28_H_21_N_6_S_2_ + H], calc. 505.1264, found: 505.1269.

1,4-bis((dipyrido[2,3-b:2′,3′-e][1,4]thiazin-10-yl)methyl)benzene (**4b**) (yield 88%), mp 300–301 °C.

^1^H NMR (DMSO_d6_) δ: ^1^H NMR (DMSO_d6_) δ: 5.12 (s, 2H, CH_2_), 6.69 (m, 1H), 7.01 (m, 2H), 7.28 (m, 2H), 8.0 (s, 1H), 8.16 (m, 2H). ^13^C NMR: 56.61, 117.05, 121.41, 122.81, 128.12, 136.10, 138,22, 140.12, 144.22, 148.81, 166.7. HRMS (EI) *m*/*z* for [C_28_H_21_N_6_S_2_ + H], calc. 505.1264, found: 505.1231.

2,6-bis((dipyrido[2,3-b:4′,3′-e][1,4]thiazin-10-yl)methyl)pyridine (**4c**) (yield 79%) mp 232–234 °C.

^1^H NMR (DMSO_d6_) δ: 5.07 (s, 4H, 2CH2), 6.14 (d, 2H), 6.48 (d, 2H), 6.57 (m, 2H), 7.01 (m, 1H, H_γpyr_), 7.5 (d, 2H), 7.86 (m, 2H), 7.95 (s, 2H, H_βpyr_), 7.99 (s, 2H). ^13^C NMR (CDCl_3_): 51.79, 109.54, 115.73, 120.44, 121.55, 133.16, 135.25, 137.82, 138.97, 144.52, 145.30, 148.38, 150.24, 154.92. HRMS (EI) *m*/*z* for [C_27_H_20_N_7_S_2_ + H], calc. 506.1216, found: 506.1274.

### 3.2. In Silico Target Prediction

Prediction of biological targets was carried out using server Way2Drug [33].

This server analyzes the biological activity of an organic drug based on the molecular recognition between individual atoms of the ligand and the molecular target. The program displays results as a percentage of the probability of a given biological activity (Pa). Based on this concept, the results obtained are helpful in SAR (*structure–activity relationships*) analysis.

### 3.3. Biological Evaluation

#### 3.3.1. Human Cancer Cell Lines

MCF7, SW480 and normal L6 muscle cells were bought from the ATCC company, USA [34]. MCF7 and SW480 adenocarcinoma cell lines were cultured, respectively, using Dulbecco’s Modified Eagle Medium (DMEM with 4.5 g/L glucose; HIMEDIA, Mumbai, India) supplemented with 100 U/mL penicillin, 100 µg/mL of streptomycin and 10% fetal bovine serum (FBS; Cat No. FBSEU500; Tico Europe, Amstelveen, The Netherlands) under standard conditions until the desired confluency was reached. Once the desired cell confluency of 70–80% was observed, the cells were sub-cultured through trypsinization, and the cell count was calculated prior to seeding in the 96-well microplate. Similarly, L6 rat myoblasts were cultured accordingly as normal cell lines to compare against MCF7 and SW480 cell lines during the MTT cell viability assay.

#### 3.3.2. MTT Cell Viability Assay

Cells were seeded in a 96-well microplate at a density of 1 × 10^4^ cells per well and were allowed to adhere to the wells overnight. Subsequently, the cells were treated with varying concentrations of 12 compounds (**1a,b,c**–**4a,b,c**) along with doxorubicin as the positive control. The cells were exposed to the tested substances at concentrations ranging from 50, 25, 10, 5 and 1 μM. The treatment was conducted over three periods of 24 h, 48 h and 72 h, respectively. After the treatment period, thiazolyl blue tetrazolium bromide (MTT) solution was added to each well at a concentration of 0.5 mg/mL and incubated for 3 h in a 37 °C incubator supplemented with 5% CO_2_. Lastly, the spent MTT medium was removed and DMSO was added to the wells, where the purple formazan crystals were solubilized, and the intensity of the purple hue was spectrophotometrically measured at 570 nm.

#### 3.3.3. Statistical Analysis

All values were represented as means ± standard deviations (n = 3). The data were statistically analyzed using GraphPad Prism 10.1.0, where the IC_50_ values of each compound treated on different cell lines at different time points were obtained by extrapolating the logarithmic dose–response curve. The IC_50_ values were further subjected to one-way ANOVA analysis along with the post hoc test to determine the statistical significance between the treatment groups and the negative control (untreated cells).

## 4. Conclusions

An efficient synthesis of a new series of dipyridothiazine dimers constructed using linkers (*o*-, *p*-xylene and 2,6-lutidine) was presented. The structure of these molecules was identified using advanced and modern two-dimensional ^1^H and ^13^C NMR spectra (COSY, NOESY, HSQC and HMBC) and HRMS mass spectrometry. In silico predictive, preliminary analysis performed using the Way2Drug server indicated anticancer, antioxidant and protective potential in degenerative diseases. Studies of the antitumor activity of the dimers were carried out in relation to two types of cancer lines: MCF7 breast cancer and SW480 colon cancer. Additionally, cytotoxicity towards normal L6 rat myoblasts was determined. Most of the obtained diazaphenothiazine derivatives showed significant antiproliferative activity and provided highly promising results. This activity depended on both the dipyridothiazine core building the dimer and the type of linker used. Moreover, the tested compounds were mostly characterized by a high selectivity index (SI). In particular, compared to the reference drug, the **4c** dimer containing 3,6-diazaphenothiazine and the lutidine system in its structure, (based on the analysis of the IC_50_ value) showed nanomolar activity towards breast cancer cells in the range of tested concentrations. Another dimer with high anticancer potential (which is three times higher than the activity of doxorubicin in both breast and colon cancer lines) is derivative **1c**, which contains 1,6-diazaphenothiazine and the lutidine system in its structure. Additionally, the compounds were characterized by low toxicity towards normal cells. The described dimers are promising anticancer agents. Further in vitro studies are planned for the most promising derivatives in order to determine the mechanism of anticancer activity related to both the internal and external apoptosis pathway, as well as the possibility of inducing necrosis.

## Figures and Tables

**Table 1:** The proton–proton and proton–carbon correlations of compound **3c**.

^1^H NMR (ppm)	NOESY	COSY	^13^C NMR (ppm)	HSQC	HMBC
CH_2_ 4.99	4.99–7.48	6.95–8.05	CH_2_ 51.89.	6.30–123.208	H_γ_ 7.84–120.44/154.90
H_9_ 6.02	4.99–6.02	6.02–7.51	C_9_ 109.53	H_9_ 6.02–109.53	H_1_ 7.48–133.16
H_4_ 6.95	4.99–7.34	7.34–7.84	C_4a_ 115.73	H_4_ 6.95–121.55	H_9_ 6.02–137.82
H_β_ 7.34	6.02–7.51		C_β_ 120.44	H_β_ 7.34–120.44	H_4_ 6.95–115.73
H_1_ 7.48	6.95–8.05		C_4_ 121.55	H_1_ 7.48–135.25	H_6_ 7.91–150.24
H_8_ 7.51			C_10a_ 133.16	H_8_ 7.51–148.38	
H_γ_ 7.84			C_1_ 135.25	H_γ_ 7.84–138.97	
H_6_ 7.91			C_9a_ 137.82	H_6_ 7.91–145.30	
H_3_ 8.05			C_γ_ 138.97	H_3_ 8.05–144.52	
			C_3_ 144.52		
			C_6_ 145.30		
			C_8_ 148.38		
			C_5a_ 150.24		
			C_α_ 154.90		

**Table 2:** Probability of activities (Pa) of dimers (**1a,b,c**–**4a,b,c**) using PASS program.

Probability of Activity Spectrum (Pa %)					
**1a**	(36%) Apoptosis antagonist	(27%) Histone deacetylase stimulant	(35%) Mitochondrial processing peptidase inhibitor	(79%) Glycosyl-phosphatidylinositol phospholipase D inhibitor	(56%) Chenodeoxy-choloyltaurine hydrolase inhibitor
**1b**	(29%) Apoptosis antagonist	(30%) Histone deacetylase stimulant	(34%) Alzheimer’s disease treatment	(80%) Glycosyl-phosphatidylinositol phospholipase D inhibitor	(58%)(S)-6-hydroxy-nicotine oxidase inhibitor
**1c**	(27%) Apoptosis antagonist	(39%) Histone deacetylase stimulant	(36%) Alzheimer’s disease treatment	(77%) Glycosyl-phosphatidylinositol phospholipase D inhibitor	(50%) Antiallergic
**2a**	(32%) Cytochrome P450 inhibitor	(63%) Anaphylatoxin receptor antagonist	(27%) p21-activated kinase 1 inhibitor	(85%) Glycosyl-phosphatidylinositol phospholipase D inhibitor	(54%) Treatment of neurodegenerative diseases
**2b**	(18%) Apoptosis antagonist	(13%) Protein kinase B alpha inhibitor	(33%) Mitochondrial processing peptidase inhibitor	(85%) Glycosyl-phosphatidylinositol phospholipase D inhibitor	(55%) Treatment of neurodegenerative diseases
**2c**	(22%) Cytochrome P450 inhibitor	(9%) Histone deacetylase stimulant	(61%) Anaphylatoxin receptor antagonist	(83%) Glycosyl-phosphatidylinositol phospholipase D inhibitor	(53%) Treatment of neurodegenerative diseases
**3a**	(43%) Cytochrome P450 inhibitor	(65%) Histone deacetylase stimulant	(30%) Mitochondrial processing peptidase inhibitor	(85%) Glycosyl-phosphatidylinositol phospholipase D inhibitor	(76%) Treatment of neurodegenerative diseases
**3b**	(40%) Cytochrome P450 inhibitor	(67%) Histone deacetylase stimulant	(43%) Alzheimer’s disease treatment	(86%) Glycosyl-phosphatidylinositol phospholipase D inhibitor	(77%) Treatment of neurodegenerative diseases
**3c**	(33%) Cytochrome P450 inhibitor	(70%) Histone deacetylase stimulant	(20%) Protein kinase inhibitor	(84%) Glycosyl-phosphatidylinositol phospholipase D inhibitor	(75%) Treatment of neurodegenerative diseases
**4a**	(29%) Cytochrome P450 inhibitor	(76%) Histone deacetylase stimulant	(60%) Alzheimer’s disease treatment	(80%) Glycosyl-phosphatidylinositol phospholipase D inhibitor	(61%) Treatment of neurodegenerative diseases
**4b**	(26%) Cytochrome P450 inhibitor	(78%) Histone deacetylase stimulant	(60%) Alzheimer’s disease treatment	(80%) Glycosyl-phosphatidylinositol phospholipase D inhibitor	(62%) Treatment of neurodegenerative diseases
**4c**	(28%) Mitochondrial processing peptidase inhibitor	(79%) Histone deacetylase stimulant	(60%) Alzheimer’s disease treatment	(77%) Glycosyl-phosphatidylinositol phospholipase D inhibitor	(60%) Treatment of neurodegenerative diseases

**Table 3:** Anticancer activities IC_50_ (μM) of dimers (**1a,b,c**–**4a,b,c**) against MCF7 breast cancer, SW480 colon cancer and normal L6 rat myoblasts.

Compounds	Anticancer Activity IC_50_ (µM)								
	24 h Treatment Period			48 h Treatment Period			72 h Treatment Period		
	L6	MCF-7	SW480	L6	MCF-7	SW480	L6	MCF-7	SW480
**1a**	20.63 ± 0.29	7.85 ± 1.67	21.84 ± 0.36	19.13 ± 1.08	7.12 ± 0.42	20.52 ± 0.79	16.14 ± 0.42	7.49 ± 1.13	17.47 ± 0.5
**1b**	15.98 ± 0.36	11.55 ± 0.15	28.65 ± 1.28	1.92± 1.39	15.92 ± 0.83	20.73 ± 1.22	11.71 ± 0.59	9.60 ± 0.46	19.76 ± 1.21
**1c**	11.28 ± 0.99	5.00 ± 1.29	0.67 ± 2.05	2.67 ± 2.13	0.75 ± 1.85	3.97 ± 1.27	6.82 ± 1.24	1.14 ± 0.51	4.27 ± 0.19
**2a**	22.06 ± 1.42	6.27 ± 0.95	9.68 ± 0.17	17.73± 0.27	9.65 ± 0.63	8.74 ± 0.03	13.04 ± 1.32	8.16 ± 0.97	7.433 ± 0.02
**2b**	25.08 ± 1.12	14.74 ± 0.86	11.89 ± 0.44	17.41 ± 2.4	12.18 ± 0.15	9.85 ± 0.18	11.95 ± 1.68	11.14 ± 1.27	3.11 ± 0.88
**2c**	17.63 ± 0.85	23.17 ± 0.33	17.24 ± 1.46	19.23± 0.13	10.02 ± 0.05	21.06 ± 0.99	17.96 ± 0.18	9.96 ± 0.03	15.92 ± 1.48
**3a**	30.82 ± 0.47	4.774 ± 0.84	6.12 ± 0.21	21.73 ± 0.48	3.17 ± 0.13	4.16 ± 0.08	21.41 ± 0.17	6.10 ± 1.33	4.56 ± 1.18
**3b**	20.83 ± 0.28	19.04 ± 1.23	24.49 ± 0.26	22.67 ± 0.29	28.03 ± 0.65	21.19 ± 0.14	22.15 ± 0.22	18.89 ± 0.15	19.30 ± 0.12
**3c**	48.98 ± 0.33	15.03 ± 0.73	22.32 ± 0.93	28.39 ± 0.37	11.74 ± 0.38	28.75 ± 1.55	35.53 ± 0.04	25.17 ± 1.74	25.95 ± 0.84
**4a**	35.90 ± 0.57	32.68 ± 2.31	22.98 ± 1.22	22.95 ± 1.39	33.77 ± 1.46	24.06 ± 0.66	14.94 ± 1.15	19.87 ± 2.36	24.40 ± 0.83
**4b**	36.74 ± 1.03	29.73 ± 1.33	29.09 ± 0.37	30.84 ± 0.93	24.24 ± 0.17	26.45 ± 0.74	11.61 ± 0.47	25.59 ± 0.35	21.41 ± 0.21
**4c**	27.05 ± 0.74	0.12 ± 1.28	16.59 ± 0.38	20.16 ± 0.75	2.112 × 10^−5^ ± 3.86	12.07 ± 0.35	13.84 ± 0.02	NA	11.59 ± 0.52
Doxorubicin	11.52 ± 0.68	28.40 ± 0.36	13.46 ± 0.11	6.62 ± 0.18	24.62 ± 2.37	10.44 ± 0.1	3.07 ± 0.38	NA	10.77 ± 0.07

NA represents IC_50_ values beyond the tested concentration range.

**Table 4:** Selectivity index (SI) of dimers (**1a,b,c**–**4a,b,c**).

Compounds	Selectivity Index (SI) of Anticancer Activity					
	24 h Treatment Period		48 h Treatment Period		72 h Treatment Period	
	MCF-7	SW480	MCF-7	SW480	MCF-7	SW480
**1a**	2.62	0.94	2.69	0.93	2.15	0.92
**1b**	1.38	0.55	0.12	0.1	1.22	0.59
**1c**	2.25	16.5	3.56	0.67	5.5	1.5
**2a**	3.5	2.32	1.83	2.03	1.59	1.75
**2b**	1.71	2.10	1.42	1.77	1.02	3.84
**2c**	0.76	1.02	1.91	0.91	1.80	1.12
**3a**	6.46	5.02	6.85	5.22	3.51	4.69
**3b**	1.09	0.95	0.81	1.07	1.17	1.15
**3c**	3.25	2.19	2.42	0.94	1.41	1.37
**4a**	1.10	1.56	0.68	0.95	0.75	0.61
**4b**	1.23	1.21	1.27	1.16	0.45	0.54
**4c**	>100	1.63	>100	1.67	-	1.34
Doxorubicin	0.41	0.85	0.25	0.63	-	0.28

## Data Availability

Data is contained within the article and Supplementary Materials.

## Supplementary Materials

The following supporting information can be downloaded at: https://www.mdpi.com/article/10.3390/molecules28227662/s1, NMR spectra and HR MS of **1a**–**4c**.
