# Supplementary material for: Synthesis and Structural Characterization of Novel Dimers of Dipyridothiazine as Promising Antiproliferative Agents
Source: Molecules. 2023 Nov 19;28(22):7662. doi: 10.3390/molecules28227662 (PMC10674446; doi:10.3390/molecules28227662)

Supplementary Material

Synthesis and Structural Characterization of Novel Dimers of Dipyridothiazine as Promising Antiproliferative Agents

Emilia Martula [1], Beata Morak-Młodawska [2*], Małgorzata Jeleń [2], Patrick N. Okechukwu [3], Abbirami Balachandran [3], Prethika Tehirunavukarasu [3], Kirthani Anamalay [3], Vaidehi Ulaganathan [4]

[1] Doctoral School of The Medical University of Silesia, Poland, d201074@365.sum.edu.pl

[2] Department of Organic Chemistry, Faculty of Pharmaceutical Sciences, The Medical University of Silesia, Jagiellońska 4, 41-200 Sosnowiec, Poland; bmlodawska@sum.edu.pl (B.M.M.); manowak@sum.edu.pl (M.J.)

[3] Department of Biotechnology, Faculty of Applied Sciences, UCSI University, Cheras, Kuala Lumpur 56000, Selangor, Malaysia; patrickn@ucsiuniversity.edu.my (P.N.O); 1001233455@ucsiuniversity.edu.my (A. B), kirthani12@hotmail.com (K. A), p rethika18@gmail.com (P. T)

[4] Department of Food Science and Nutrition, Faculty of Applied Sciences, UCSI University, Cheras, Kuala Lumpur 56000, Selangor, Malaysia; vaidehi@ucsiuniversity.edu.my (V.U)

Content

1. NMR spectra and HR MS of 1,2-bis((dipyrido[2,3-b:2',3'-e][1,4]thiazin-10-yl)methyl)benzene (**1a**) 2
2. NMR spectra and HR MS of 1,4-bis((dipyrido[2,3-b:2',3'-e][1,4]thiazin-10-yl)methyl)benzene (**1b**) 6
3. NMR spectra and HR MS of 2,6-bis((dipyrido[2,3-b:2',3'-e][1,4]thiazin-10-yl)methyl)pyridine (**1c**) 9
4. NMR spectra and HR MS of 1,2-bis((dipyrido[3,2-b:3',4'-e][1,4]thiazin-10-yl)methyl)benzene (**2a**) 11
5. NMR spectra and HR MS of 1,4-bis((dipyrido[2,3-b:2',3'-e][1,4]thiazin-10-yl)methyl)benzene (**2b**) 14
6. NMR spectra and HR MS of 2,6-bis((dipyrido[3,2-b:3',4`-e][1,4]thiazin-10-yl)methyl)pyridine (**2c**)
7. NMR spectra and HR MS of 1,2-bis((dipyrido[3,4-b:3',4'-e][1,4]thiazin-10-yl)methyl)benzene (**3a**) 20
8. NMR spectra and HR MS of 1,4-bis((dipyrido[2,3-b:2',3'-e][1,4]thiazin-10-yl)methyl)benzene (**3b**) 21

9. NMR spectra and HR MS of 2,6-bis((dipyrido[3,4-b:3',4'-e][1,4]thiazin-10-yl)methyl)pyridine (**3c**) 26

^{1}H NMR of 2,6-bis((dipyrido[3,4-b:3',4'-e][1,4]thiazin-10-yl)methyl)pyridine (**3c**) 26

COSY NMR of 2,6-bis((dipyrido[3,4-b:3',4'-e][1,4]thiazin-10-yl)methyl)pyridine (**3c**) 27

NOESY NMR of 2,6-bis((dipyrido[3,4-b:3',4'-e][1,4]thiazin-10-yl)methyl)pyridine (**3c**) 28

^{13}C NMR of 2,6-bis((dipyrido[3,4-b:3',4'-e][1,4]thiazin-10-yl)methyl)pyridine (**3c**) 29

HSQC NMR of 2,6-bis((dipyrido[3,4-b:3',4'-e][1,4]thiazin-10-yl)methyl)pyridine (**3c**) 31

HMBC NMR of 2,6-bis((dipyrido[3,4-b:3',4'-e][1,4]thiazin-10-yl)methyl)pyridine (**3c**) 32

HR MS of 2,6-bis((dipyrido[3,4-b:3',4'-e][1,4]thiazin-10-yl)methyl)pyridine (**3c**)

10. NMR spectra and HR MS of 1,2-bis((dipyrido[2,3-b:4',3'-e][1,4]thiazin-5-yl)methyl)benzene (**4a**) 34

11. NMR spectra and HR MS of 1,4-bis((dipyrido[2,3-b:2',3'-e][1,4]thiazin-10-yl)methyl)benzene (**4b**) 37

12. NMR spectra and HR MS of 2,6-bis((dipyrido[2,3-b:4',3'-e][1,4]thiazin-10-yl)methyl)pyridine (**4c**) 39

NMR spectra and HR MS of 1,2-bis((dipyrido[2,3-b:2',3'-e][1,4]thiazin-10-yl)methyl)benzene (**1a**)

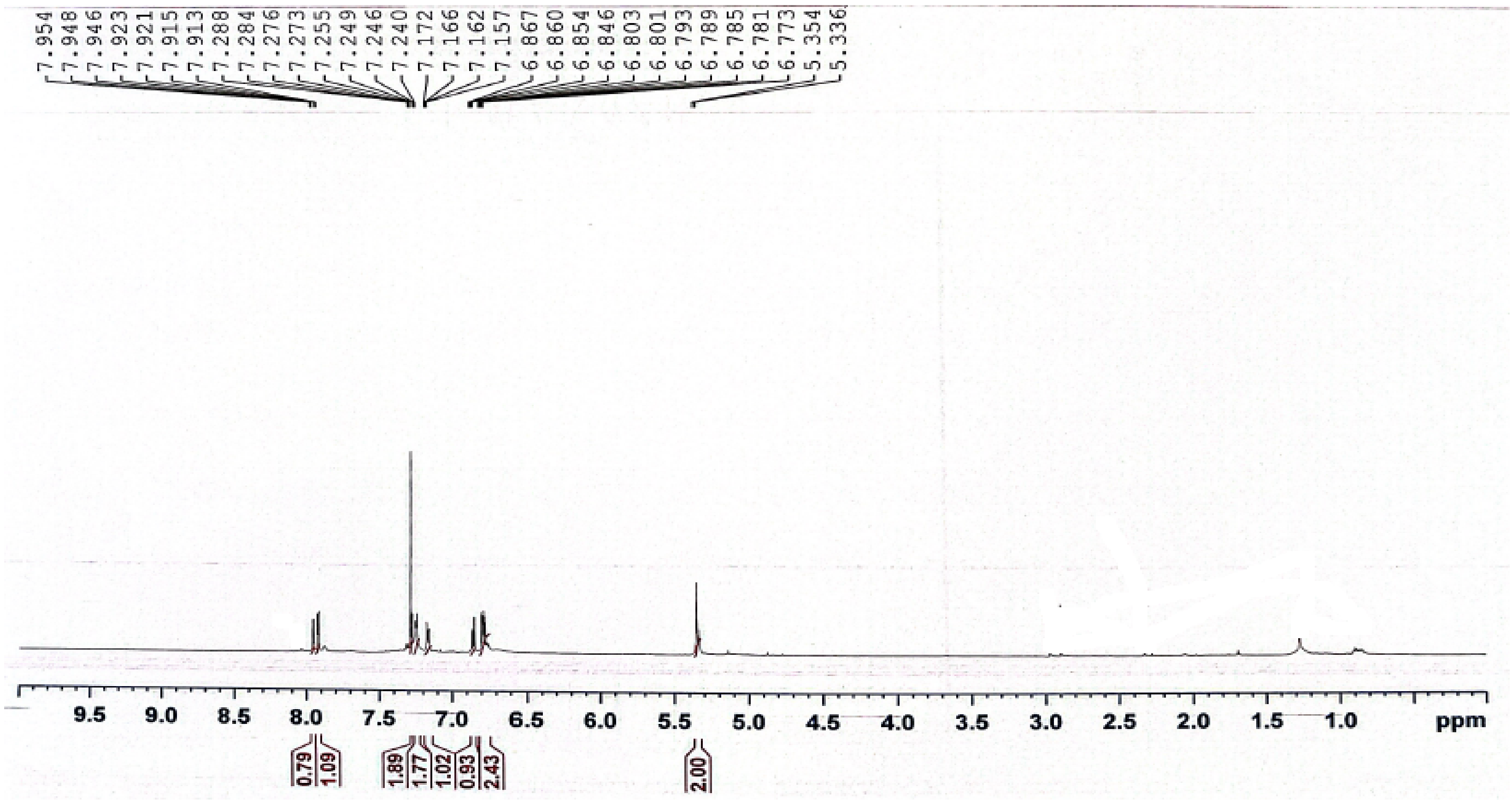

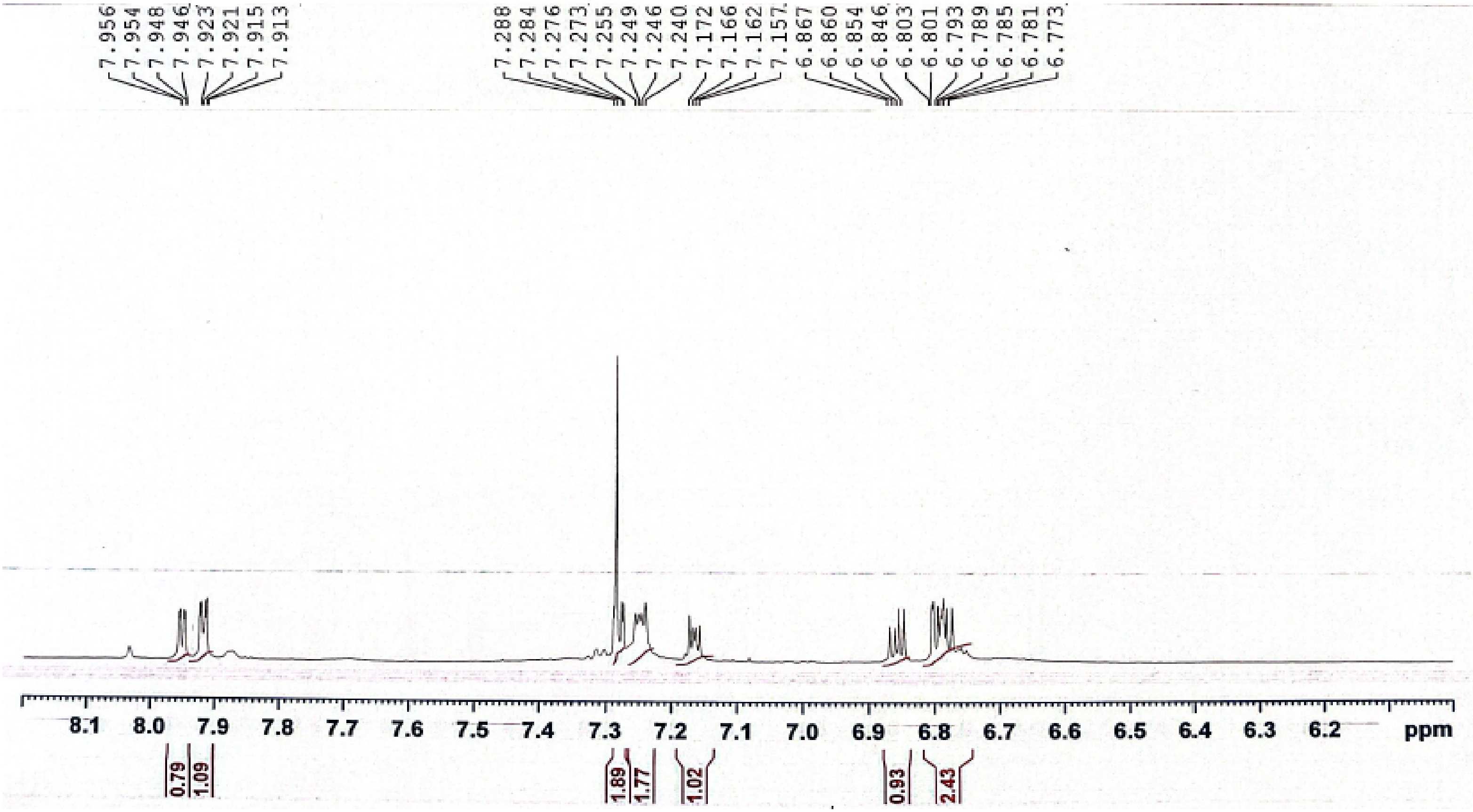
7.956
7.954
7.948
7.946
7.923
7.921
7.915
7.913
7.288
7.284
7.276
7.273
7.255
7.249
7.246
7.240
7.172
7.166
7.162
7.157
6.867
6.860
6.854
6.846
6.803
6.801
6.793
6.789
6.785
6.781
6.773
8.1 8.0 7.9 7.8 7.7 7.6 7.5 7.4 7.3 7.2 7.1 7.0 6.9 6.8 6.7 6.6 6.5 6.4 6.3 6.2 ppm
0.79
1.09
1.89
1.77
1.02
0.93
2.43

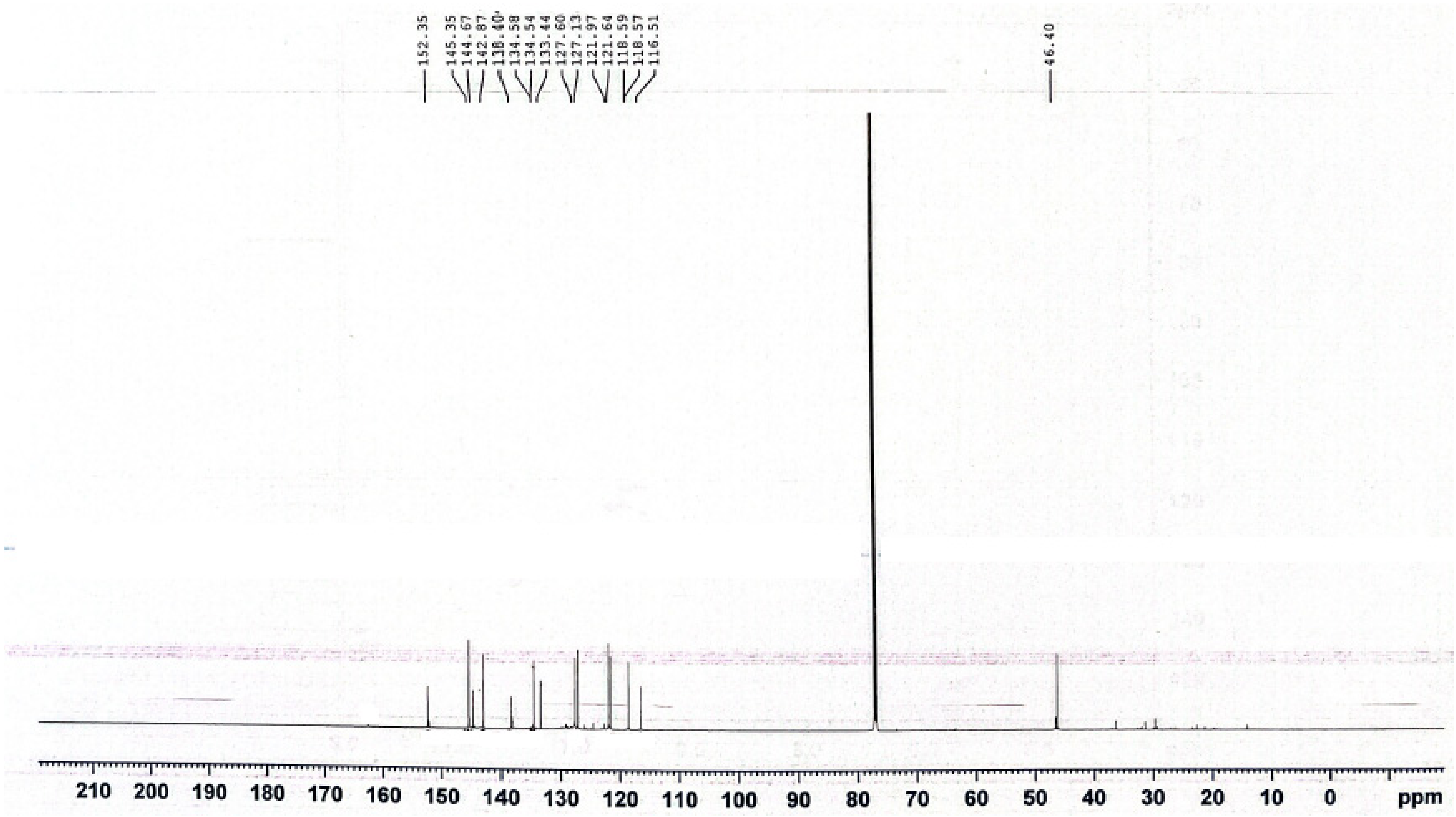
152.35
145.35
144.67
142.87
138.40
134.58
134.54
133.44
127.60
127.13
121.97
121.64
118.59
118.57
116.51
46.40
210 200 190 180 170 160 150 140 130 120 110 100 90 80 70 60 50 40 30 20 10 0 ppm

Acquisition Parameter

Source Type	ESI	Ion Polarity	Positive	Set Nebulizer	0.3 Bar
Focus	Active	Set Capillary	4000 V	Set Dry Heater	200 °C
Scan Begin	100 m/z	Set End Plate Offset	-500 V	Set Dry Gas	3.0 l/min
Scan End	1000 m/z	Set Charging Voltage	2000 V	Set Divert Valve	Source
		Set Corona	0 nA	Set APCI Heater	0 °C

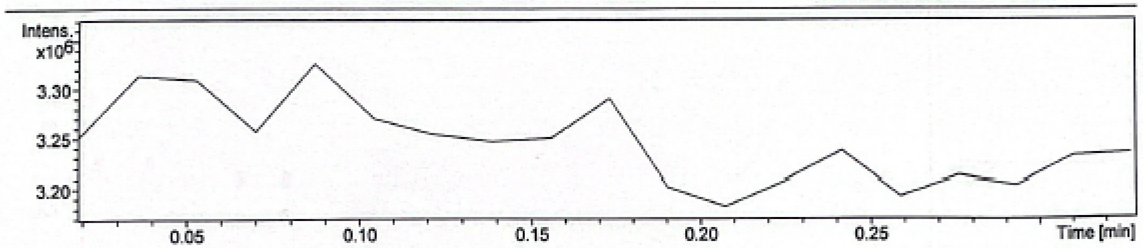

+MS, 0.1-0.2min #3-11

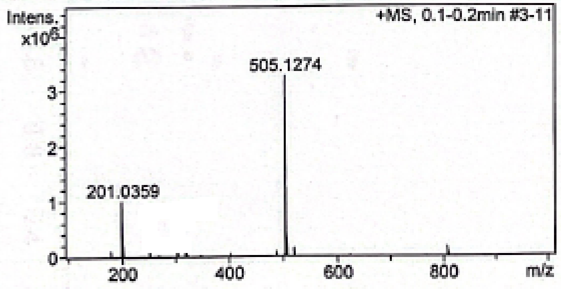

NMR spectra and HR MS of 1,4-bis((dipyrido[2,3-b:2',3'-e][1,4]thiazin-10-yl)methyl)benzene (**1b**)

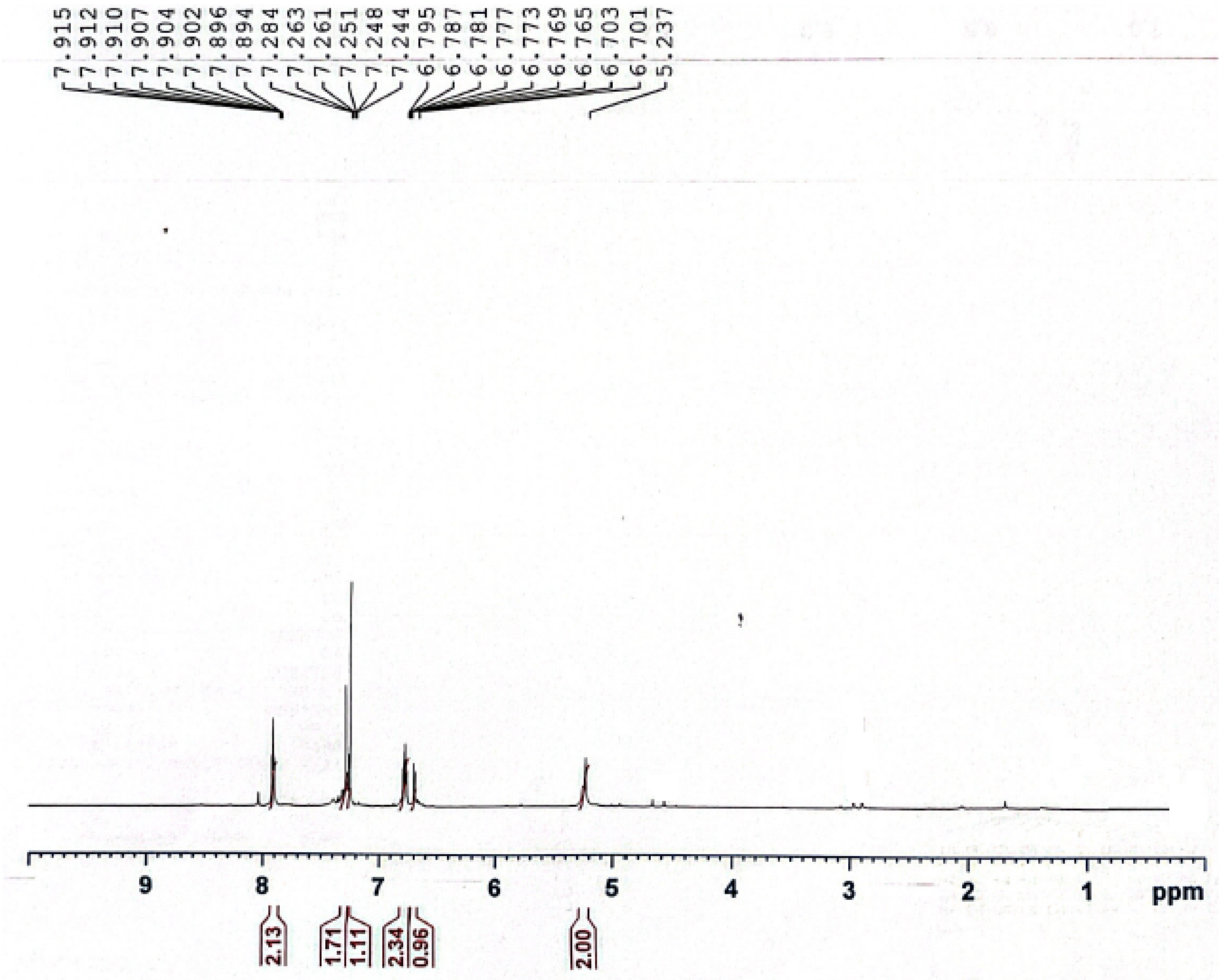

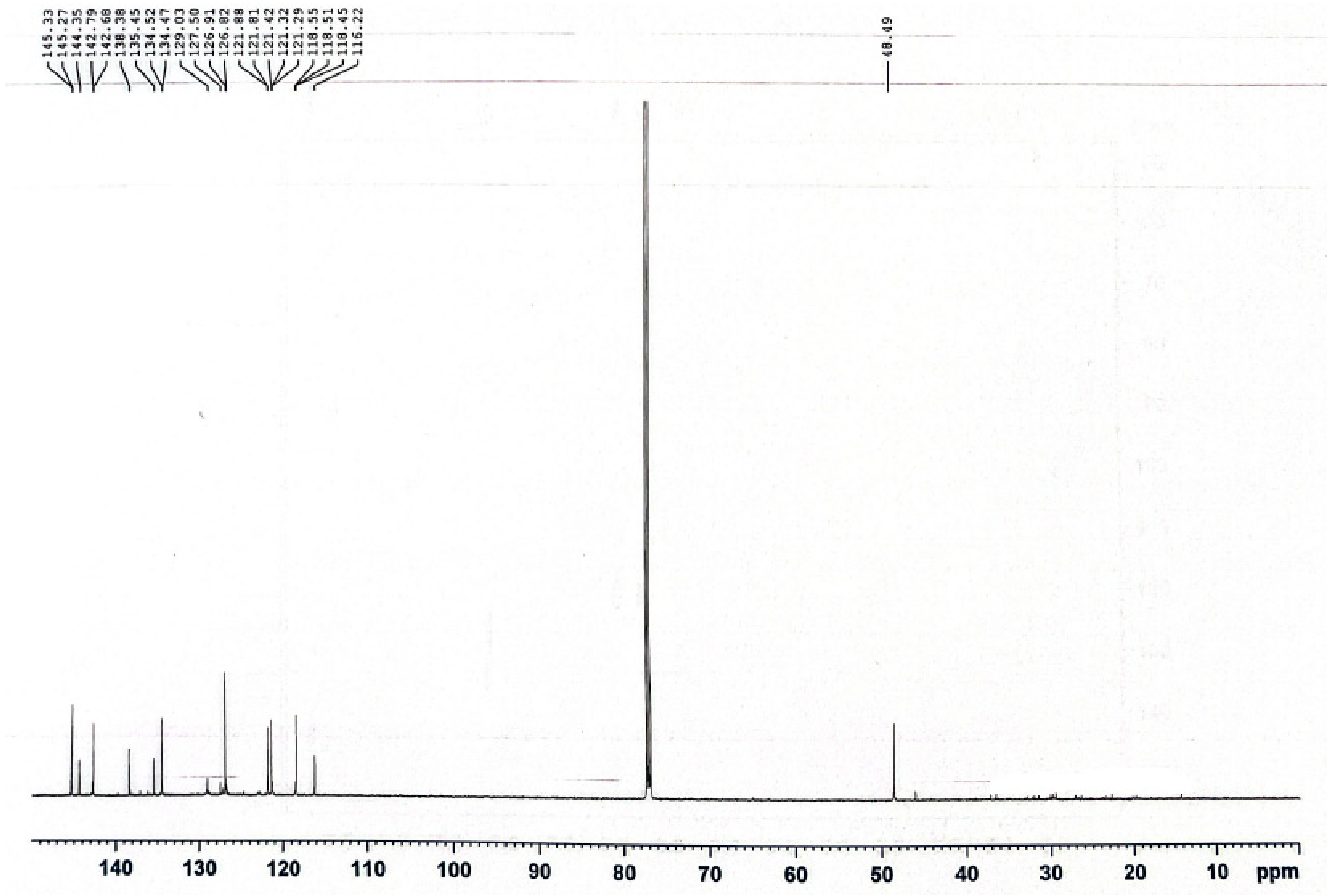
145.33
145.27
144.35
142.79
142.68
138.38
135.45
134.52
134.47
129.03
127.50
126.91
126.82
121.88
121.81
121.42
121.32
121.29
118.55
118.51
118.45
116.22
48.49
140
130
120
110
100
90
80
70
60
50
40
30
20
10
ppm

Acquisition Parameter

Source Type	ESI	Ion Polarity	Positive	Set Nebulizer	0.3 Bar
Focus	Active	Set Capillary	4000 V	Set Dry Heater	200 °C
Scan Begin	100 m/z	Set End Plate Offset	-500 V	Set Dry Gas	3.0 l/min
Scan End	1000 m/z	Set Charging Voltage	2000 V	Set Divert Valve	Source
		Set Corona	0 nA	Set APCI Heater	0 °C

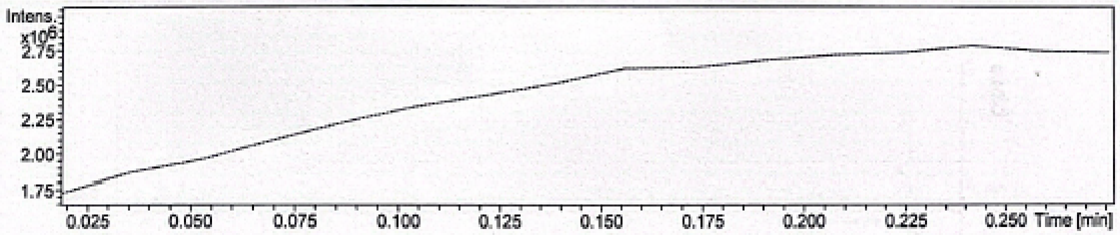

+MS, 0.0-0.2min #2-9

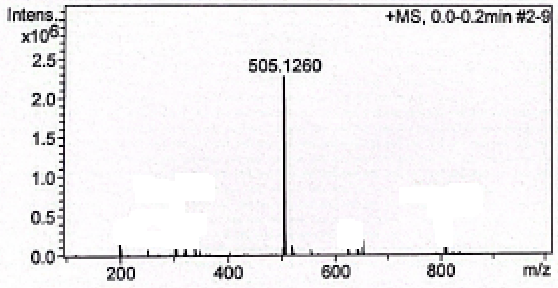

NMR spectra and HR MS of 2,6-bis((dipyrido[2,3-b:2',3'-e][1,4]thiazin-10-yl)methyl)pyridine (**1c**)

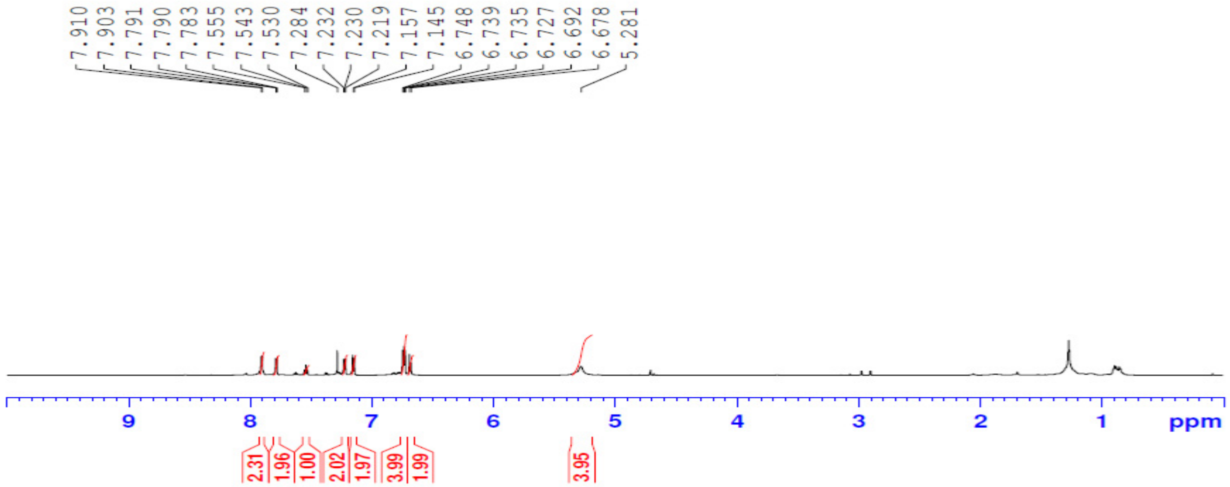

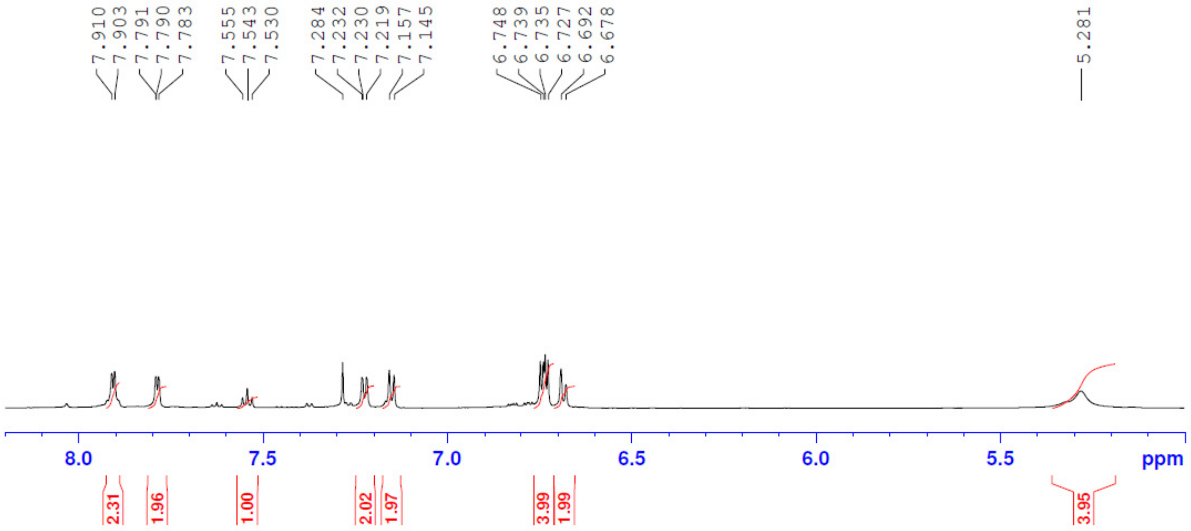

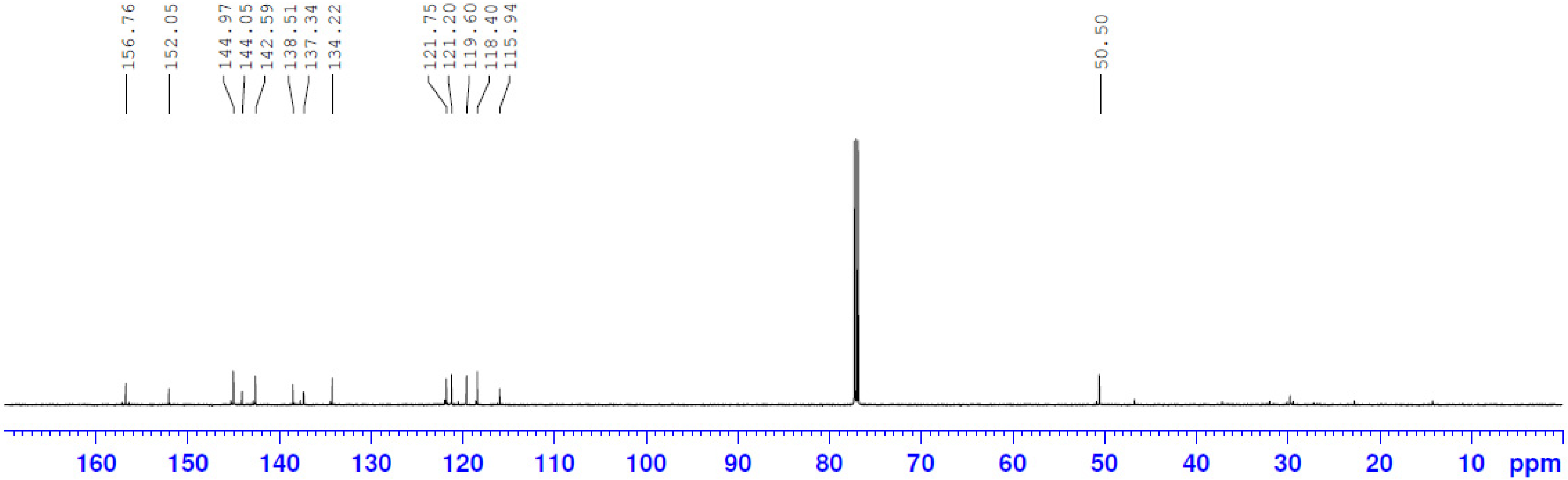
156.76
152.05
144.97
144.05
142.59
138.51
137.34
134.22
121.75
121.20
119.60
118.40
115.94
50.50
160 150 140 130 120 110 100 90 80 70 60 50 40 30 20 10 ppm

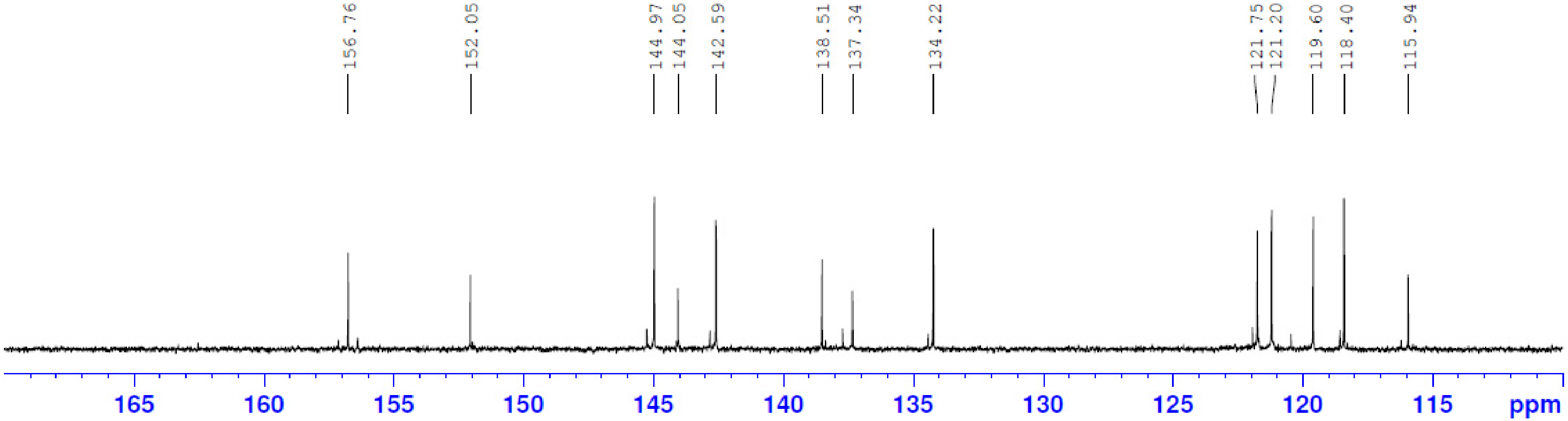
156.76
152.05
144.97
144.05
142.59
138.51
137.34
134.22
121.75
121.20
119.60
118.40
115.94
165 160 155 150 145 140 135 130 125 120 115 ppm

NMR spectra and HR MS of 1,2-bis((dipyrido[3,2-b:3',4'-e][1,4]thiazin-10-yl)methyl)benzene (**2a**)

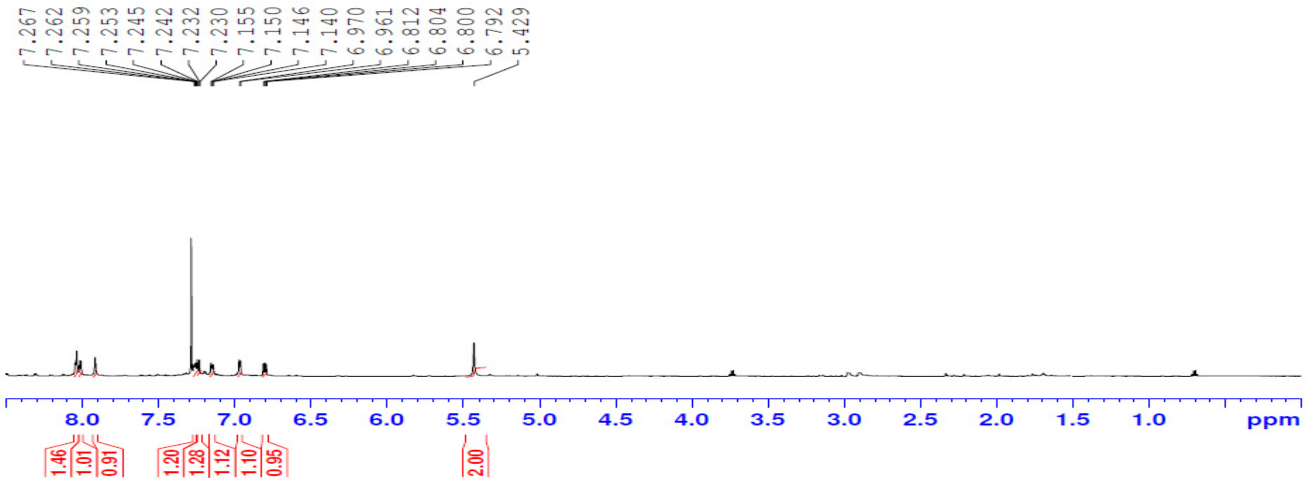

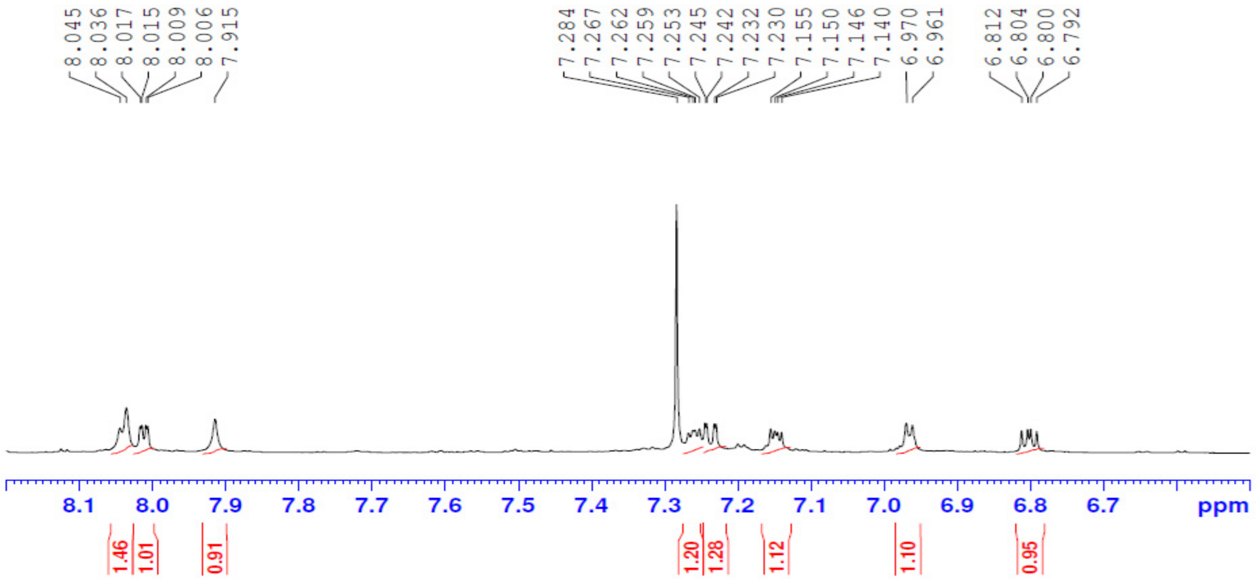

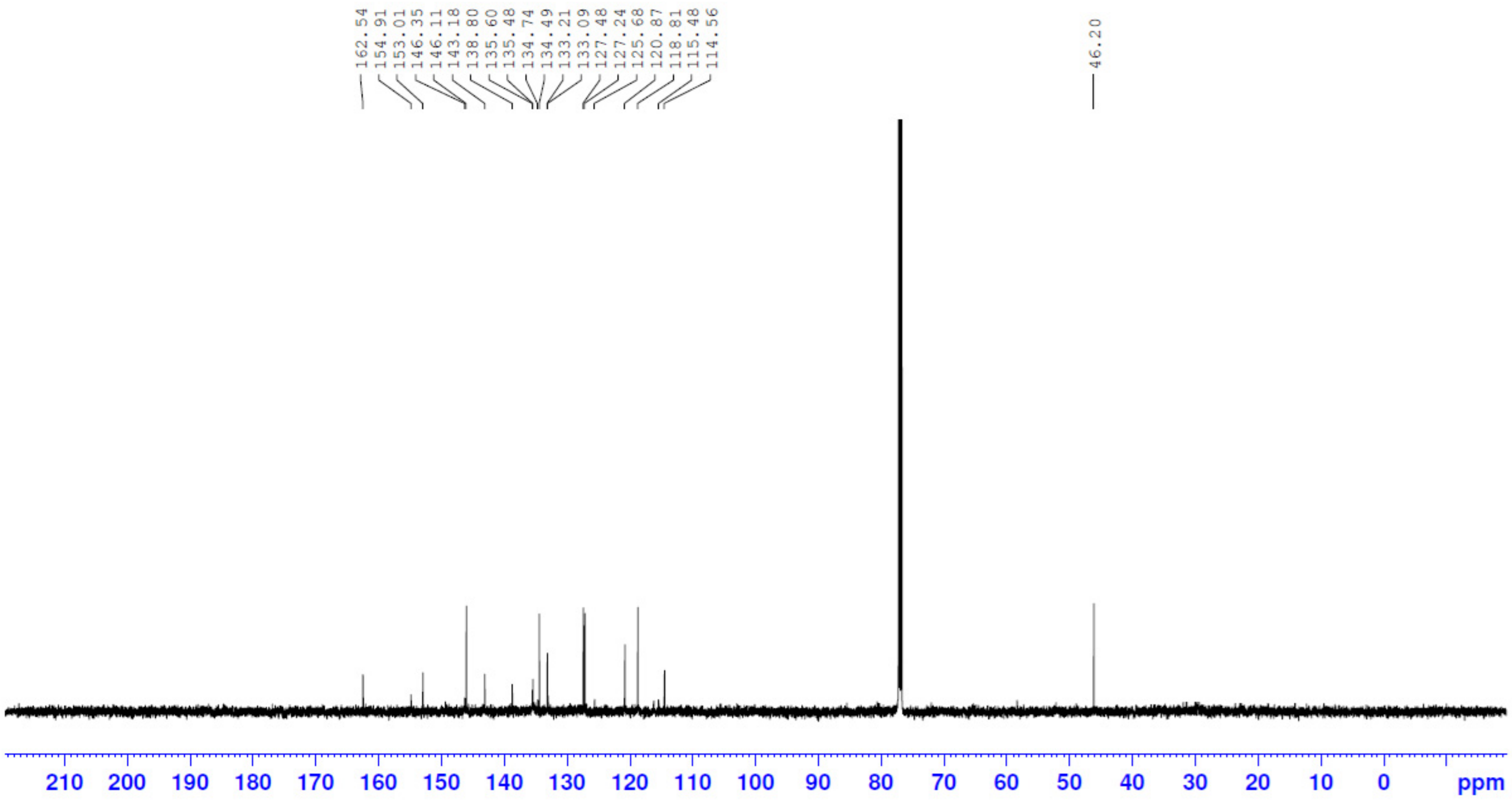
162.54
154.91
153.01
146.35
146.11
143.18
138.80
135.60
135.48
134.74
134.49
133.21
133.09
127.48
127.24
125.68
120.87
118.81
115.48
114.56
46.20
210 200 190 180 170 160 150 140 130 120 110 100 90 80 70 60 50 40 30 20 10 0 ppm

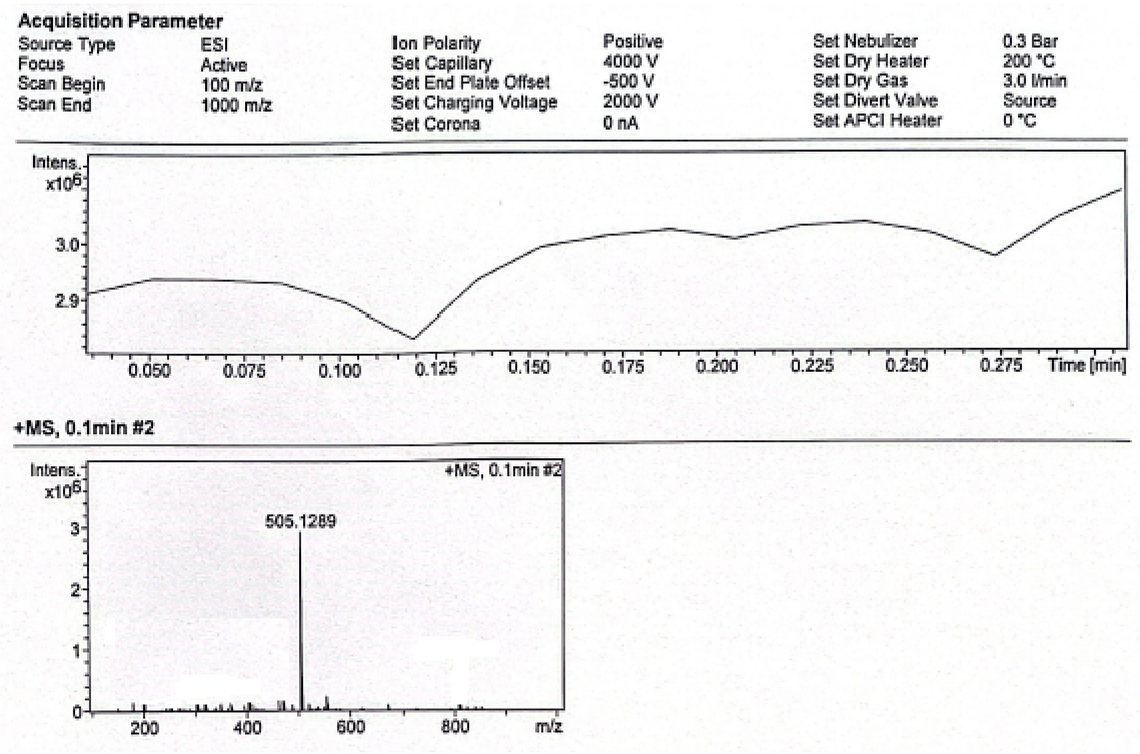
Acquisition Parameter
Source Type ESI
Focus Active
Scan Begin 100 m/z
Scan End 1000 m/z
Ion Polarity Positive
Set Capillary 4000 V
Set End Plate Offset -500 V
Set Charging Voltage 2000 V
Set Corona 0 nA
Set Nebulizer 0.3 Bar
Set Dry Heater 200 °C
Set Dry Gas 3.0 l/min
Set Divert Valve Source
Set APCI Heater 0 °C
Intens. x10^6
3.0
2.9
0.050
0.075
0.100
0.125
0.150
0.175
0.200
0.225
0.250
0.275
Time [min]
+MS, 0.1min #2
Intens. x10^6
+MS, 0.1min #2
505.1289
3
2
1
0
200
400
600
800
m/z

NMR spectra and HR MS of 1,4-bis((dipyrido[2,3-b:2',3'-e][1,4]thiazin-10-yl)methyl)benzene (**2b**)

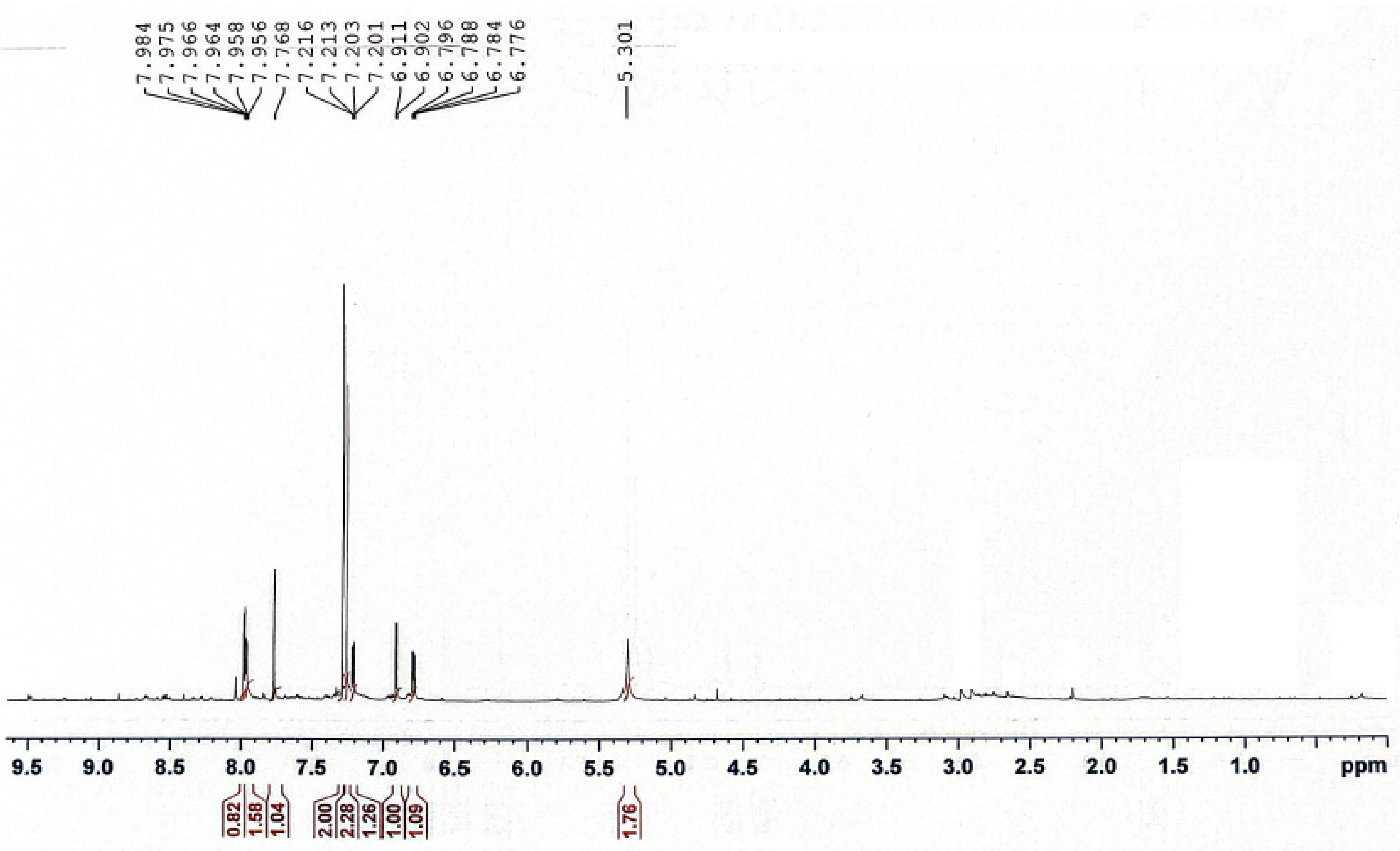

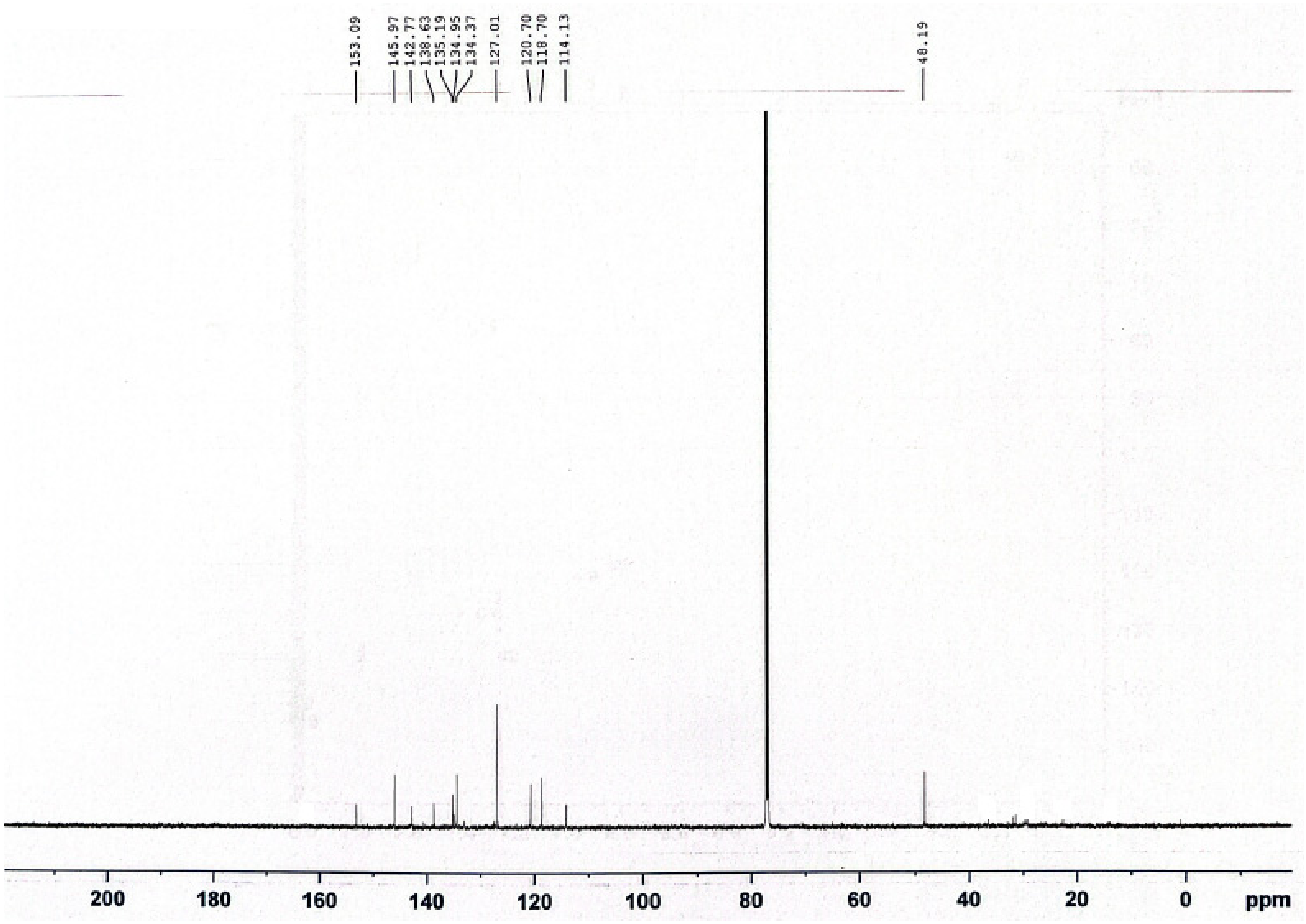
153.09
145.97
142.77
138.63
135.19
134.95
134.37
127.01
120.70
118.70
114.13
48.19
200
180
160
140
120
100
80
60
40
20
0
ppm

Acquisition Parameter

Source Type	ESI	Ion Polarity	Positive	Set Nebulizer	0.3 Bar
Focus	Active	Set Capillary	4000 V	Set Dry Heater	200 °C
Scan Begin	100 m/z	Set End Plate Offset	-500 V	Set Dry Gas	3.0 l/min
Scan End	1000 m/z	Set Charging Voltage	2000 V	Set Divert Valve	Source
		Set Corona	0 nA	Set APCI Heater	0 °C

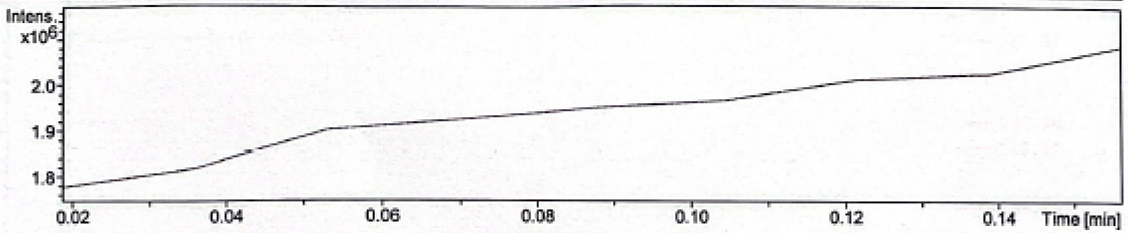

+MS, 0.0-0.1min #2-8

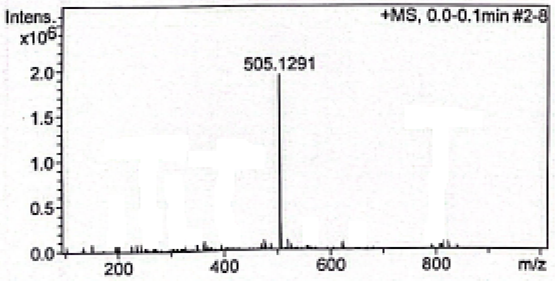

NMR spectra and HR MS of 2,6-bis((dipyrido[3,2-b:3',4`-e][1,4]thiazin-10-yl)methyl)pyridine (**2c**)

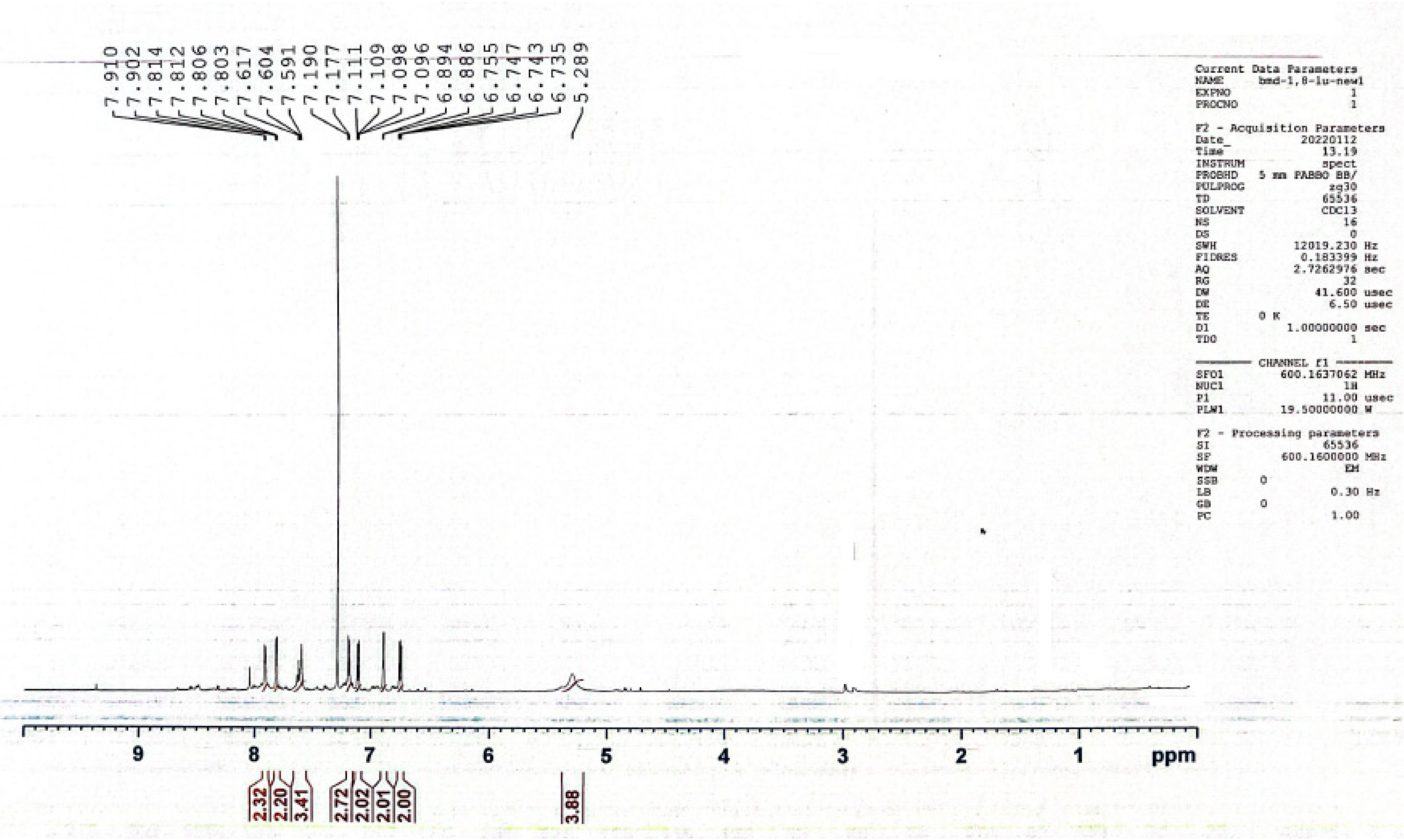

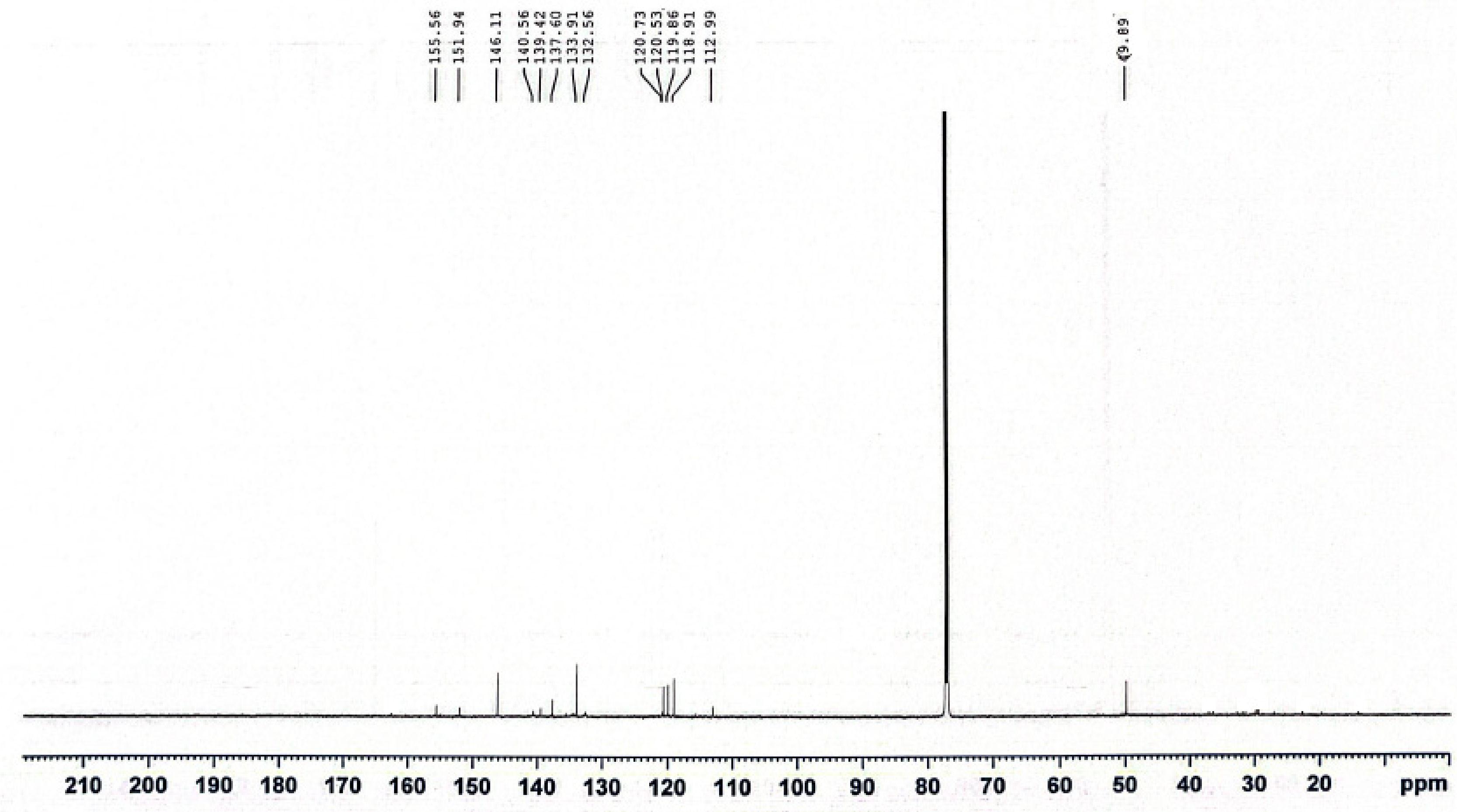
155.56
151.94
146.11
140.56
139.42
137.60
133.91
132.56
120.73
120.53
119.86
118.91
112.99
49.89
210 200 190 180 170 160 150 140 130 120 110 100 90 80 70 60 50 40 30 20 ppm

Acquisition Parameter

Source Type	ESI	Ion Polarity	Positive	Set Nebulizer	0.3 Bar
Focus	Active	Set Capillary	4000 V	Set Dry Heater	200 °C
Scan Begin	100 m/z	Set End Plate Offset	-500 V	Set Dry Gas	3.0 l/min
Scan End	1000 m/z	Set Charging Voltage	2000 V	Set Divert Valve	Source
		Set Corona	0 nA	Set APCI Heater	0 °C

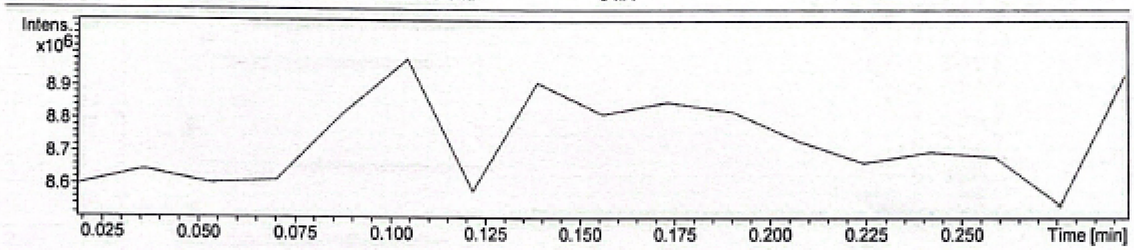

+MS, 0.0-0.1min #2-3

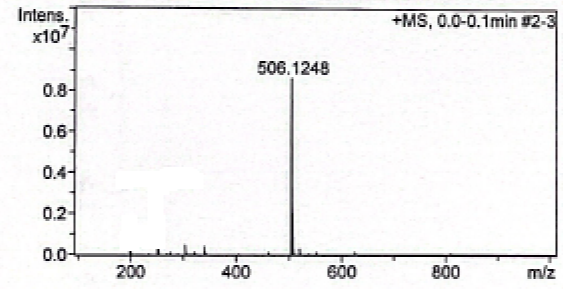

NMR spectra and HR MS of 1,2-bis((dipyrido[3,4-b:3',4'-e][1,4]thiazin-10-yl)methyl)benzene (**3a**)

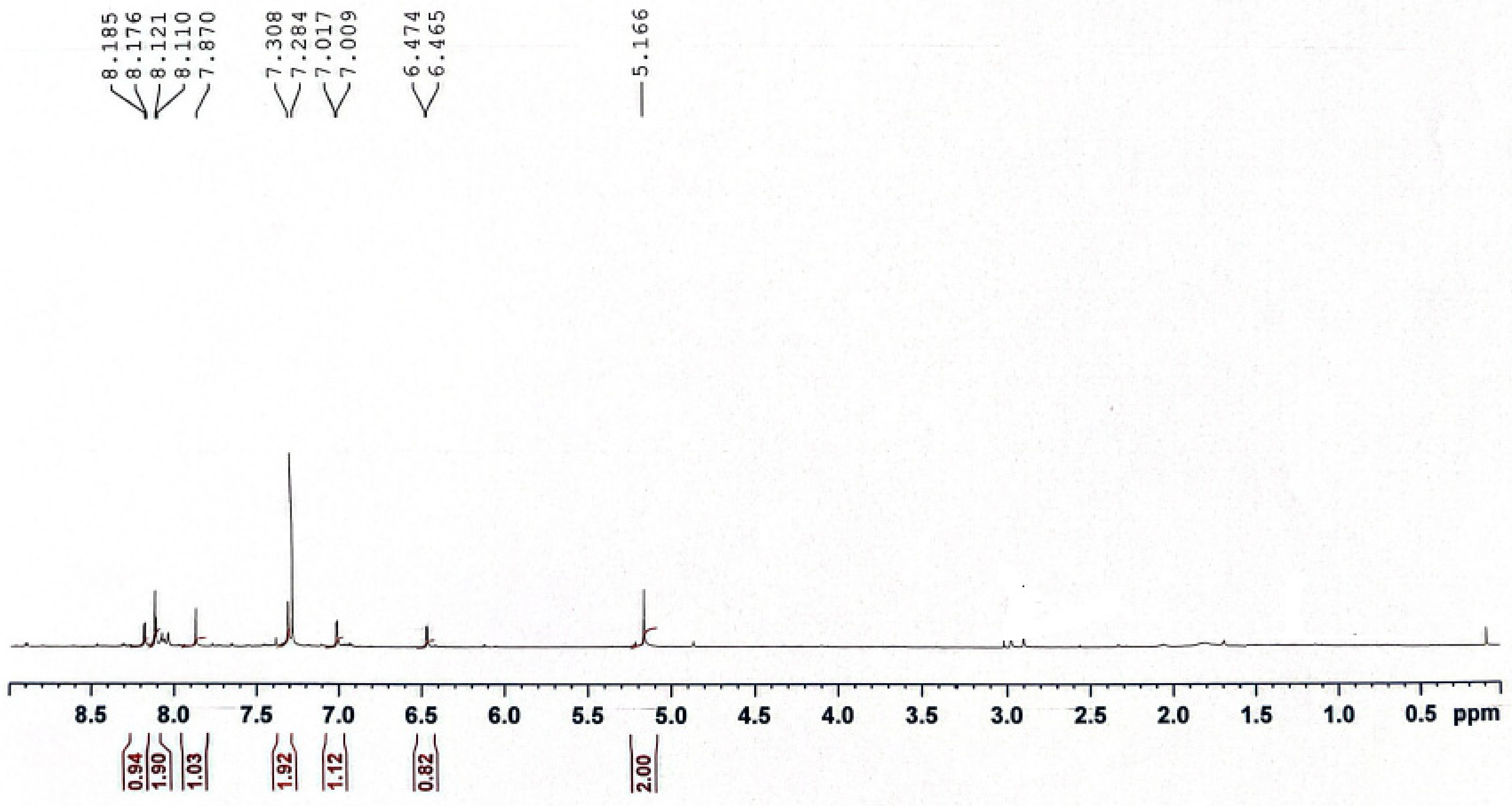

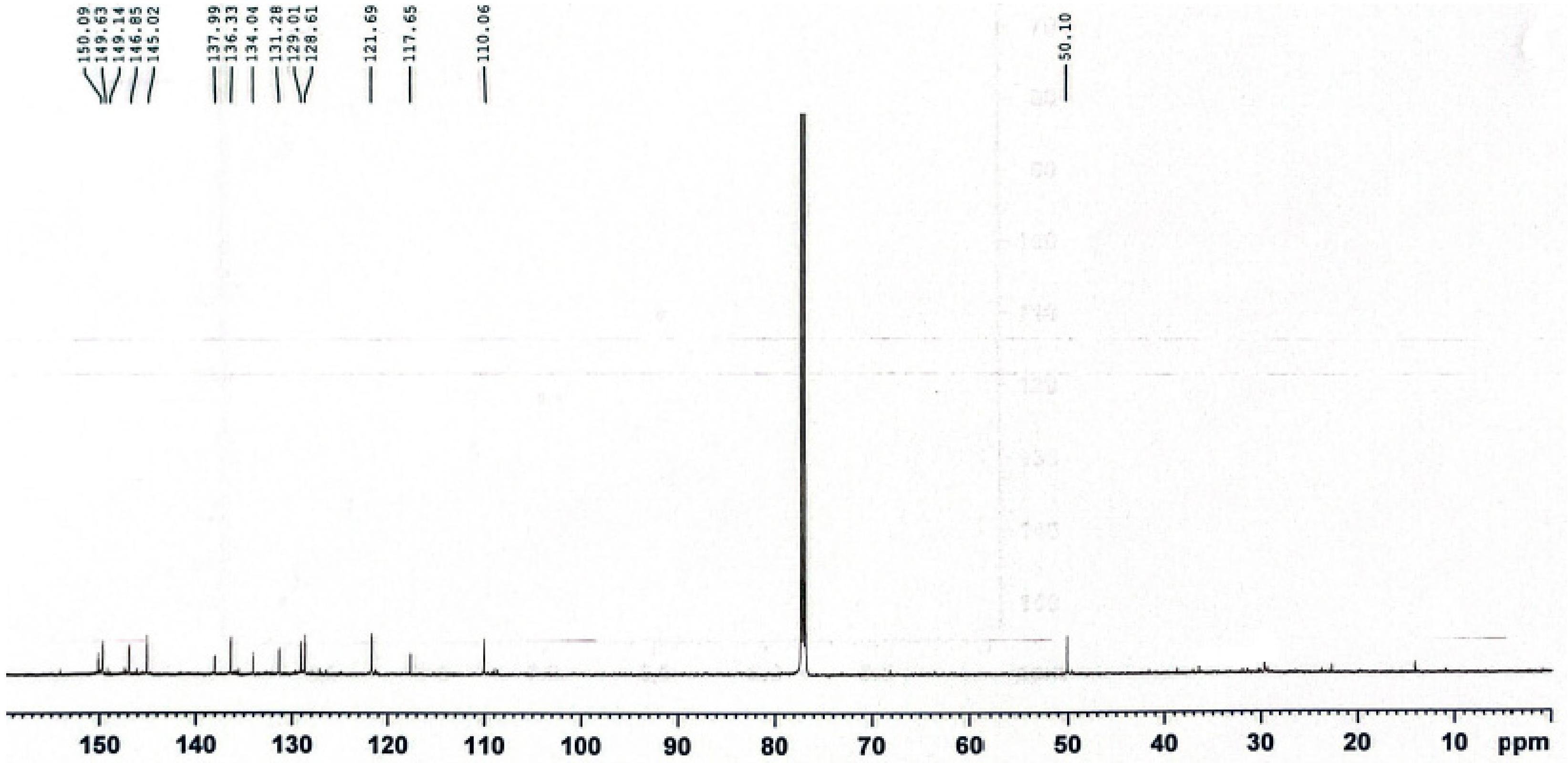
150.09
149.63
149.14
146.85
145.02
137.99
136.33
134.04
131.28
129.01
128.61
121.69
117.65
110.06
50.10
150 140 130 120 110 100 90 80 70 60 50 40 30 20 10 ppm

Acquisition Parameter

Source Type	ESI	Ion Polarity	Positive	Set Nebulizer	0.3 Bar
Focus	Active	Set Capillary	4000 V	Set Dry Heater	200 °C
Scan Begin	100 m/z	Set End Plate Offset	-500 V	Set Dry Gas	3.0 l/min
Scan End	1000 m/z	Set Charging Voltage	2000 V	Set Divert Valve	Source
		Set Corona	0 nA	Set APCI Heater	0 °C

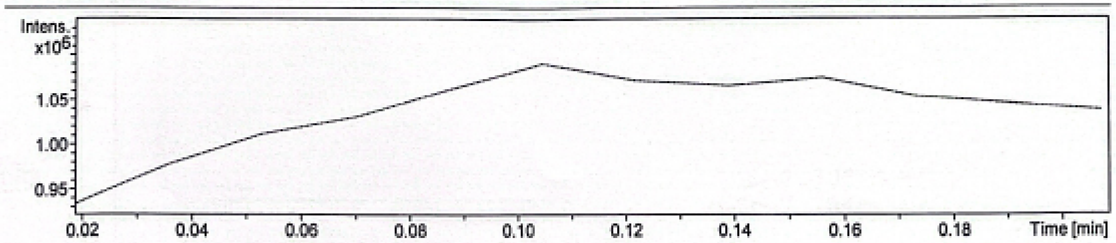

+MS, 0.0-0.1min #2-8

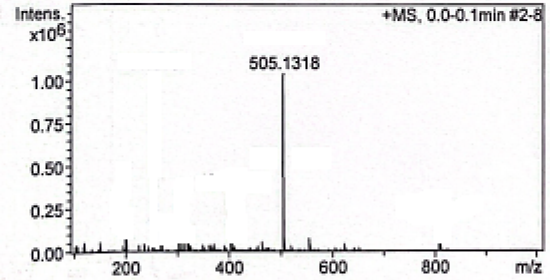

NMR spectra and HR MS of 1,4-bis((dipyrido[2,3-b:2',3'-e][1,4]thiazin-10-yl)methyl)benzene (**3b**)

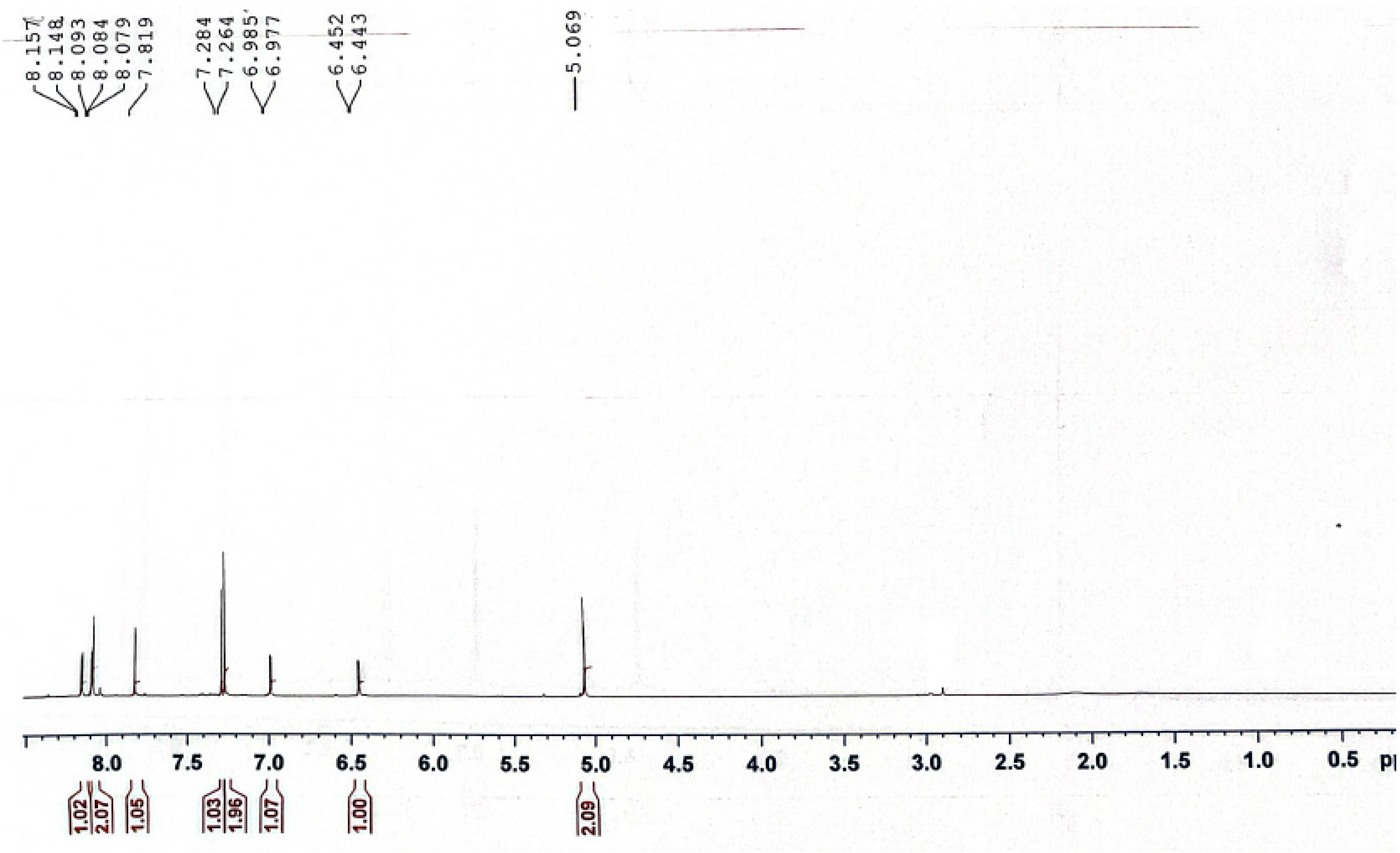

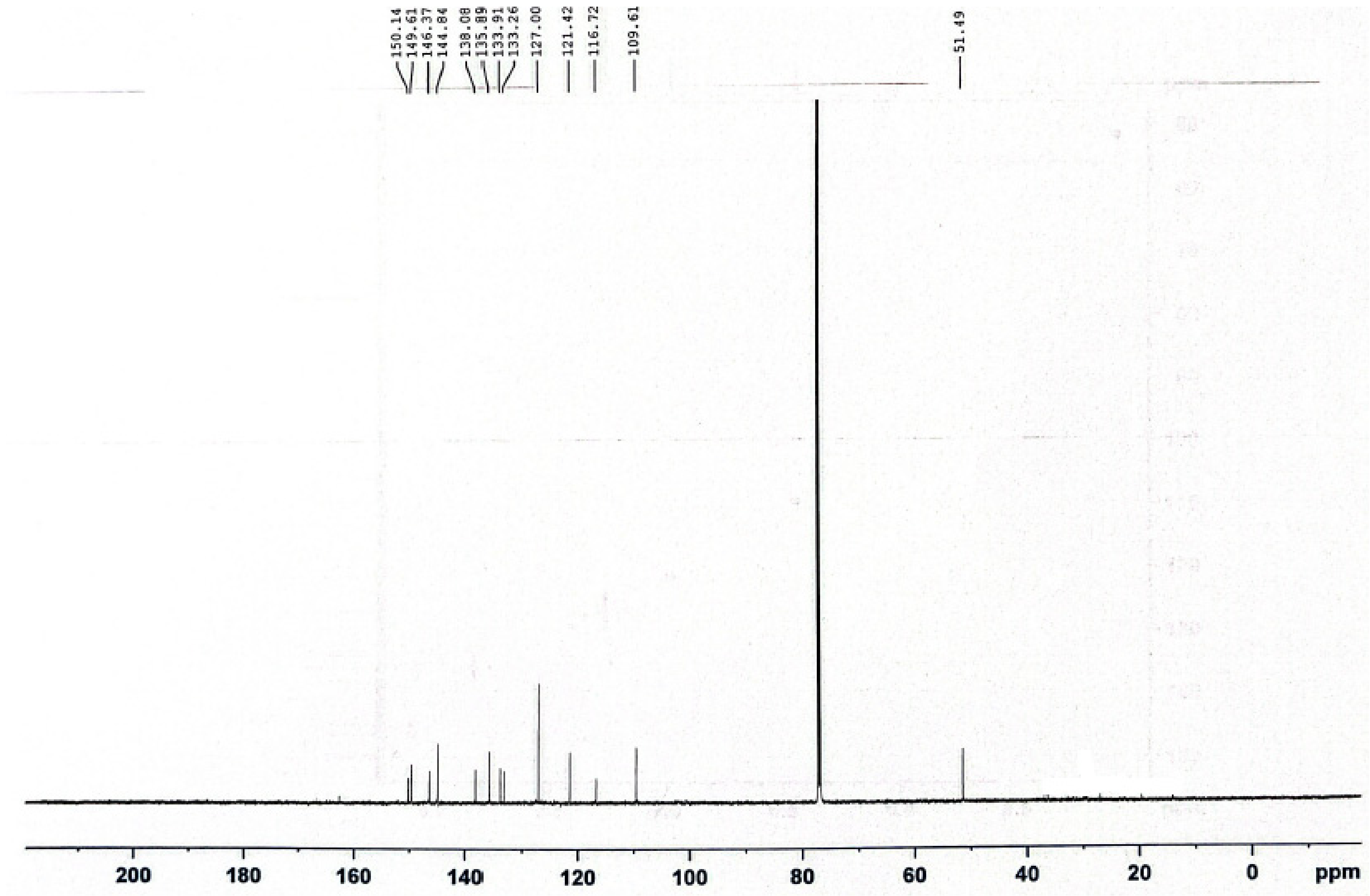
150.14
149.61
146.37
144.84
138.08
135.89
133.91
133.26
127.00
121.42
116.72
109.61
51.49
200
180
160
140
120
100
80
60
40
20
0
ppm

Acquisition Parameter

Source Type	ESI	Ion Polarity	Positive	Set Nebulizer	0.3 Bar
Focus	Active	Set Capillary	4000 V	Set Dry Heater	200 °C
Scan Begin	100 m/z	Set End Plate Offset	-500 V	Set Dry Gas	3.0 l/min
Scan End	1000 m/z	Set Charging Voltage	2000 V	Set Divert Valve	Source
		Set Corona	0 nA	Set APCI Heater	0 °C

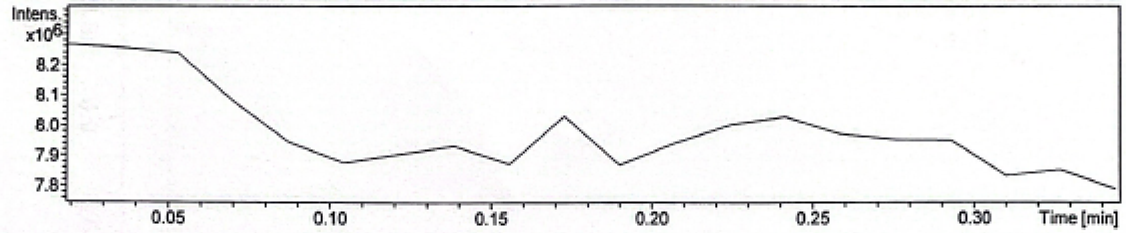

+MS, 0.0-0.3min #2-19

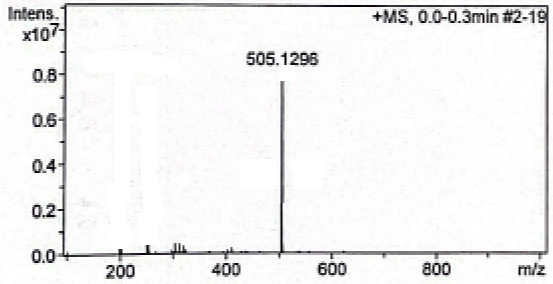

NMR spectra and HR MS of 2,6-bis((dipyrido[3,4-b:3',4'-e][1,4]thiazin-10-yl)methyl)pyridine (**3c**)

. ^{1}H NMR of 2,6-bis((dipyrido[3,4-b:3',4'-e][1,4]thiazin-10-yl)methyl)pyridine (**3c**)

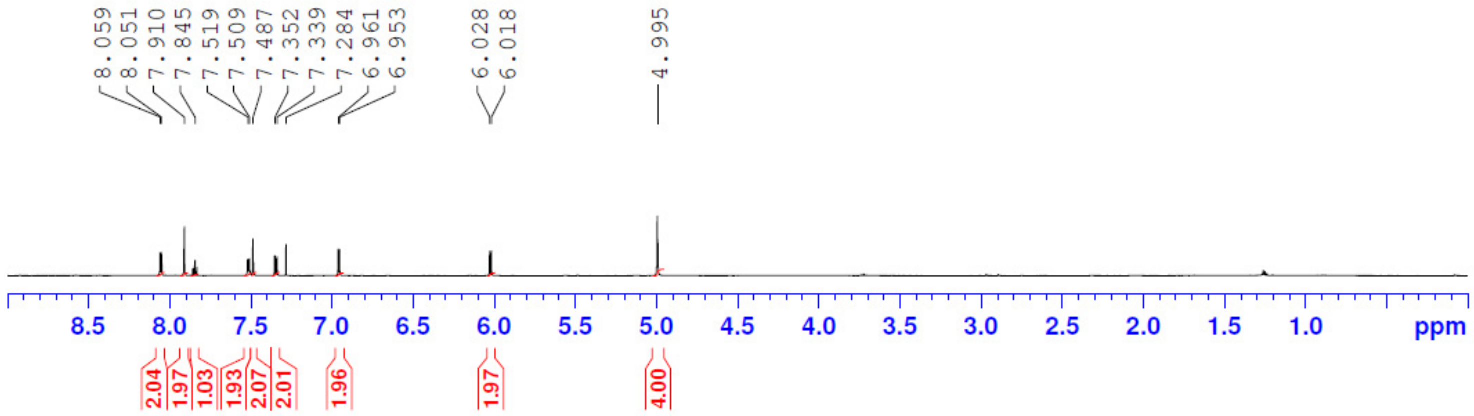

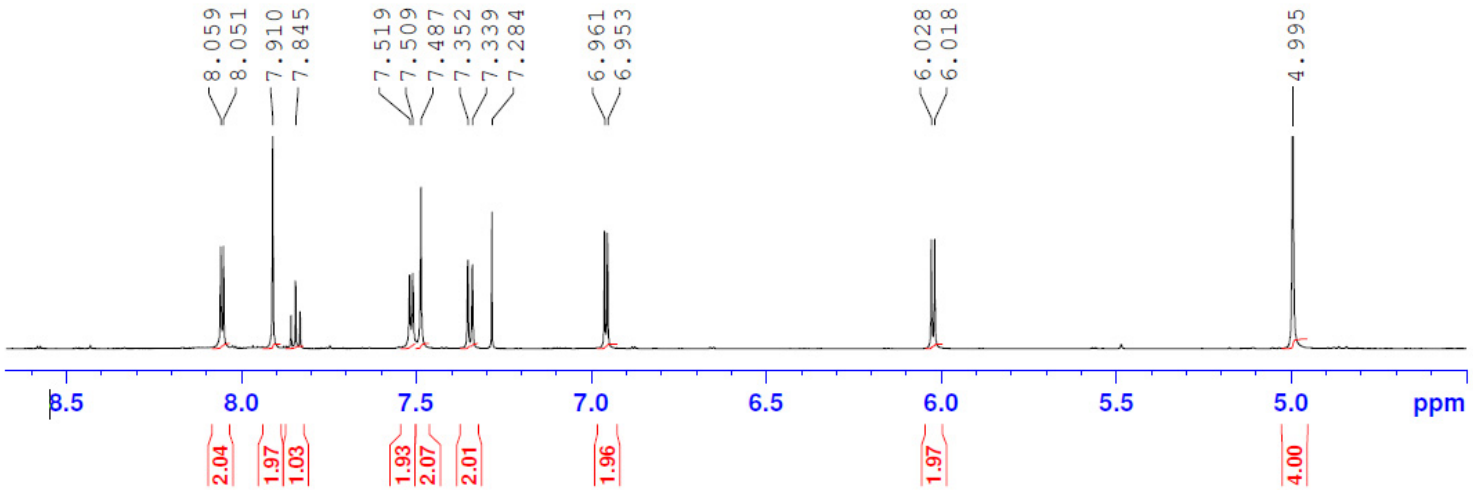

COSY NMR of 2,6-bis((dipyrido[3,4-b:3',4'-e][1,4]thiazin-10-yl)methyl)pyridine (**3c**)

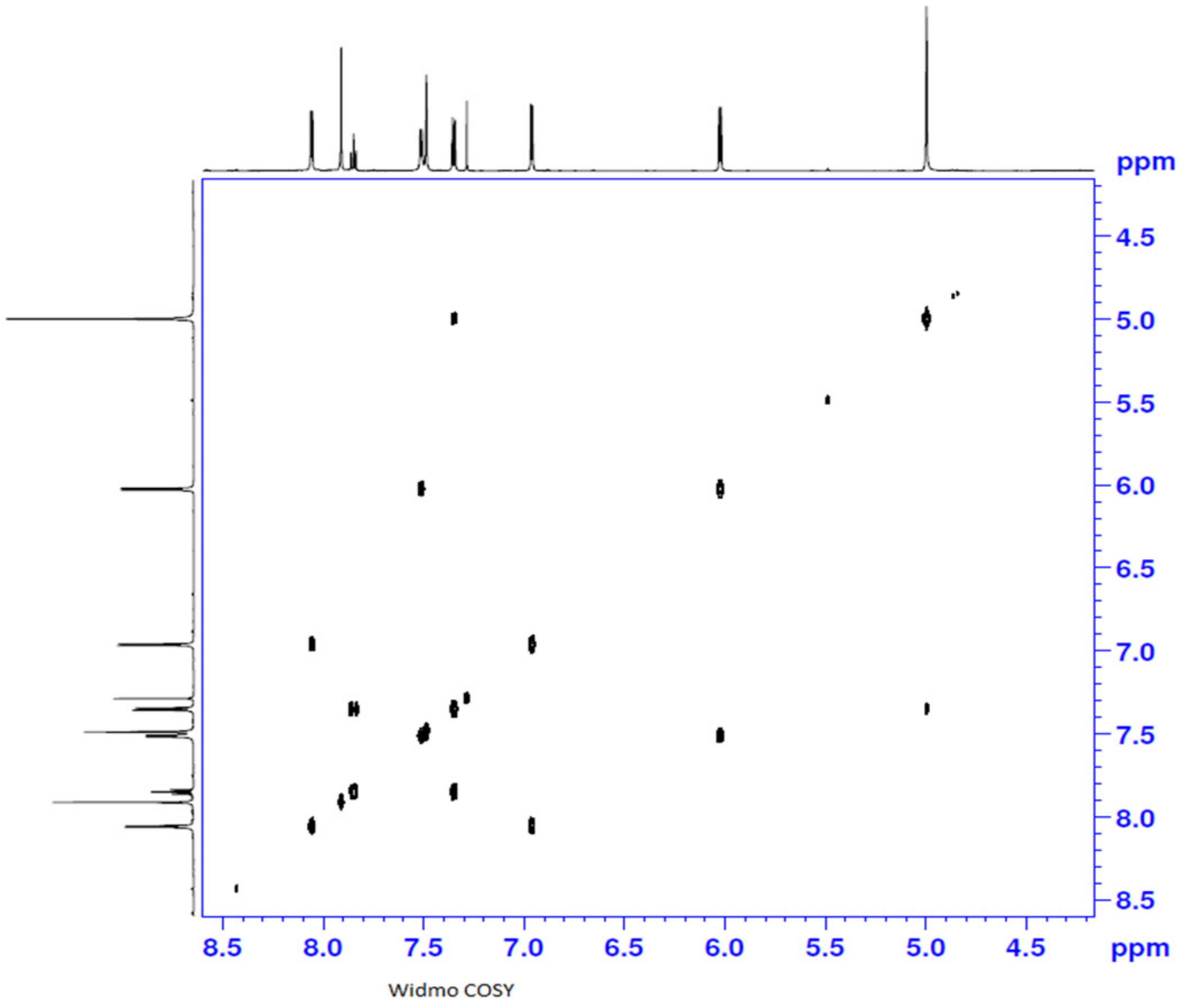

NOESY NMR of 2,6-bis((dipyrido[3,4-b:3',4'-e][1,4]thiazin-10-yl)methyl)pyridine (**3c**)

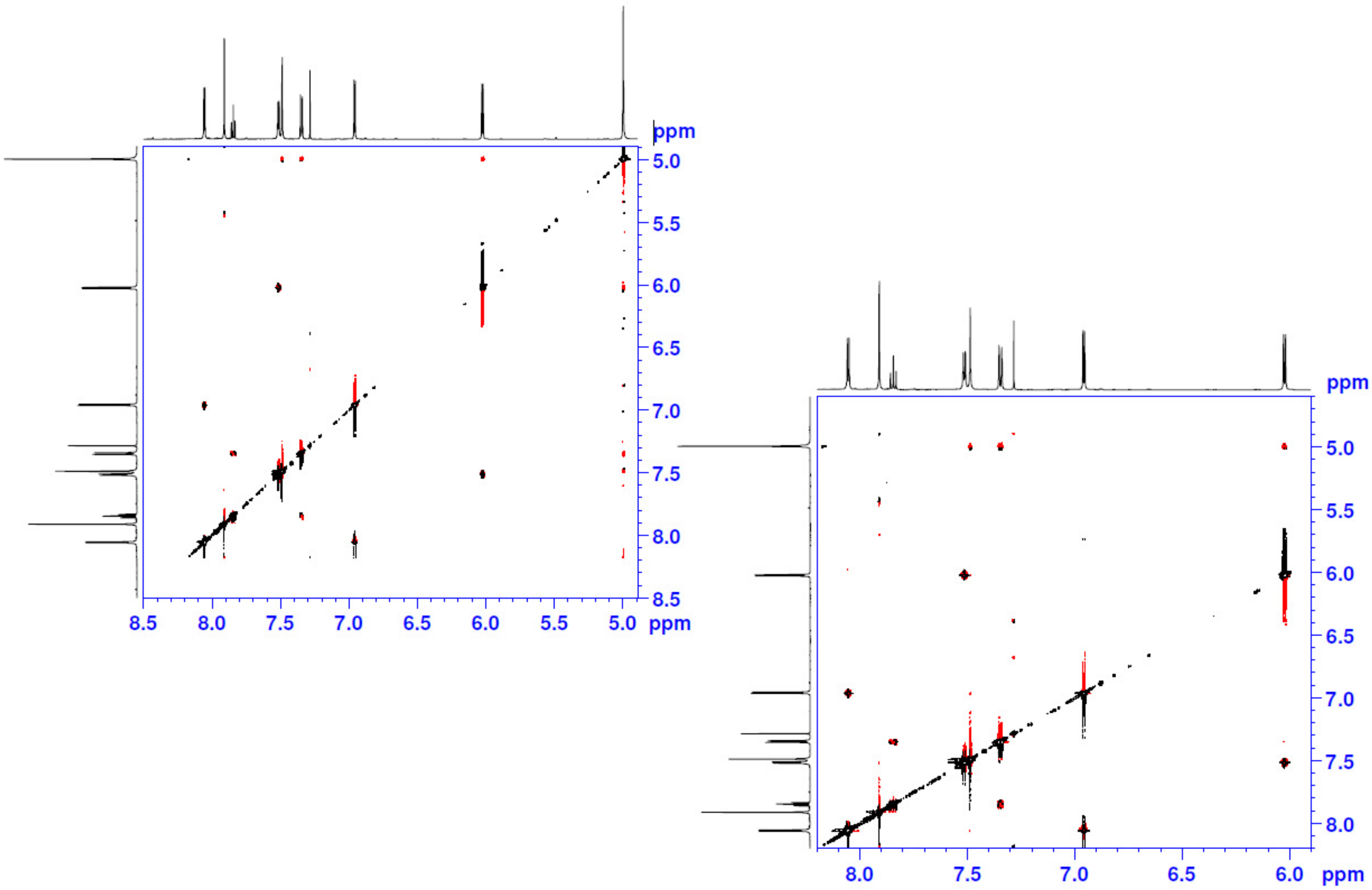

^{13}C NMR of 2,6-bis((dipyrido[3,4-b:3',4'-e][1,4]thiazin-10-yl)methyl)pyridine (**3c**)

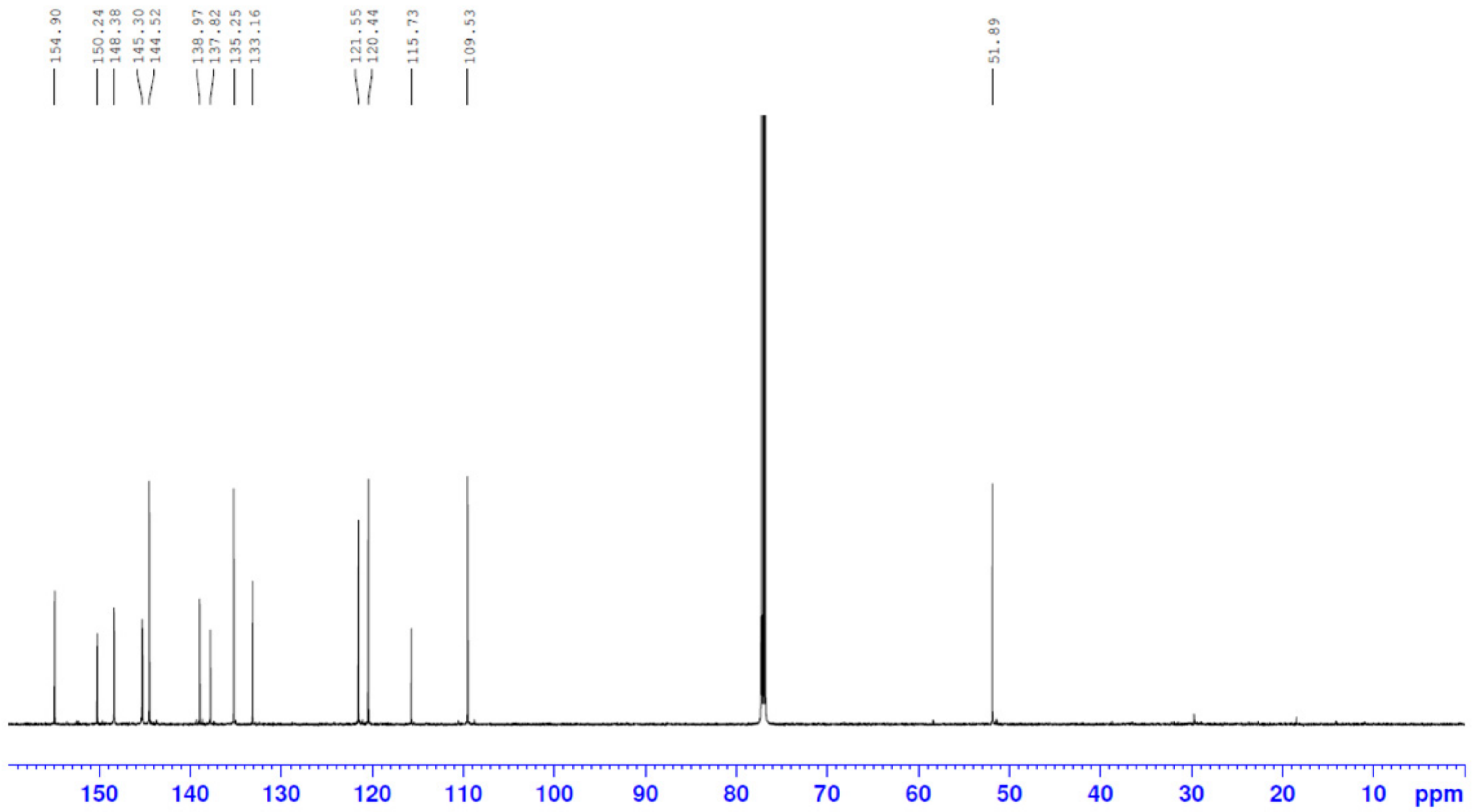

^{13}C NMR of 2,6-bis((dipyrido[3,4-b:3',4'-e][1,4]thiazin-10-yl)methyl)pyridine (**3c**) – aromatic part

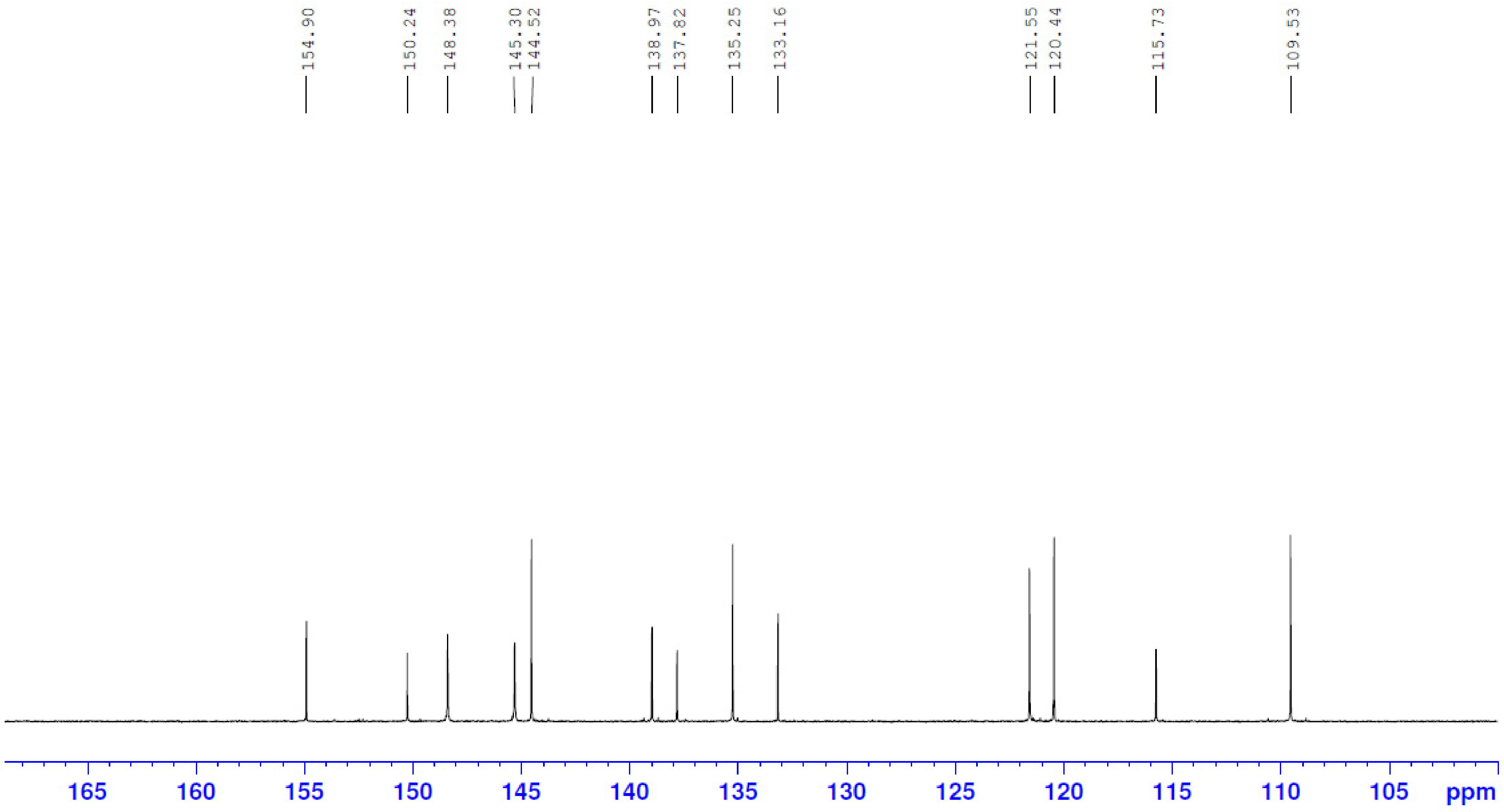

HSQC NMR of 2,6-bis((dipyrido[3,4-b:3',4'-e][1,4]thiazin-10-yl)methyl)pyridine (**3c**)

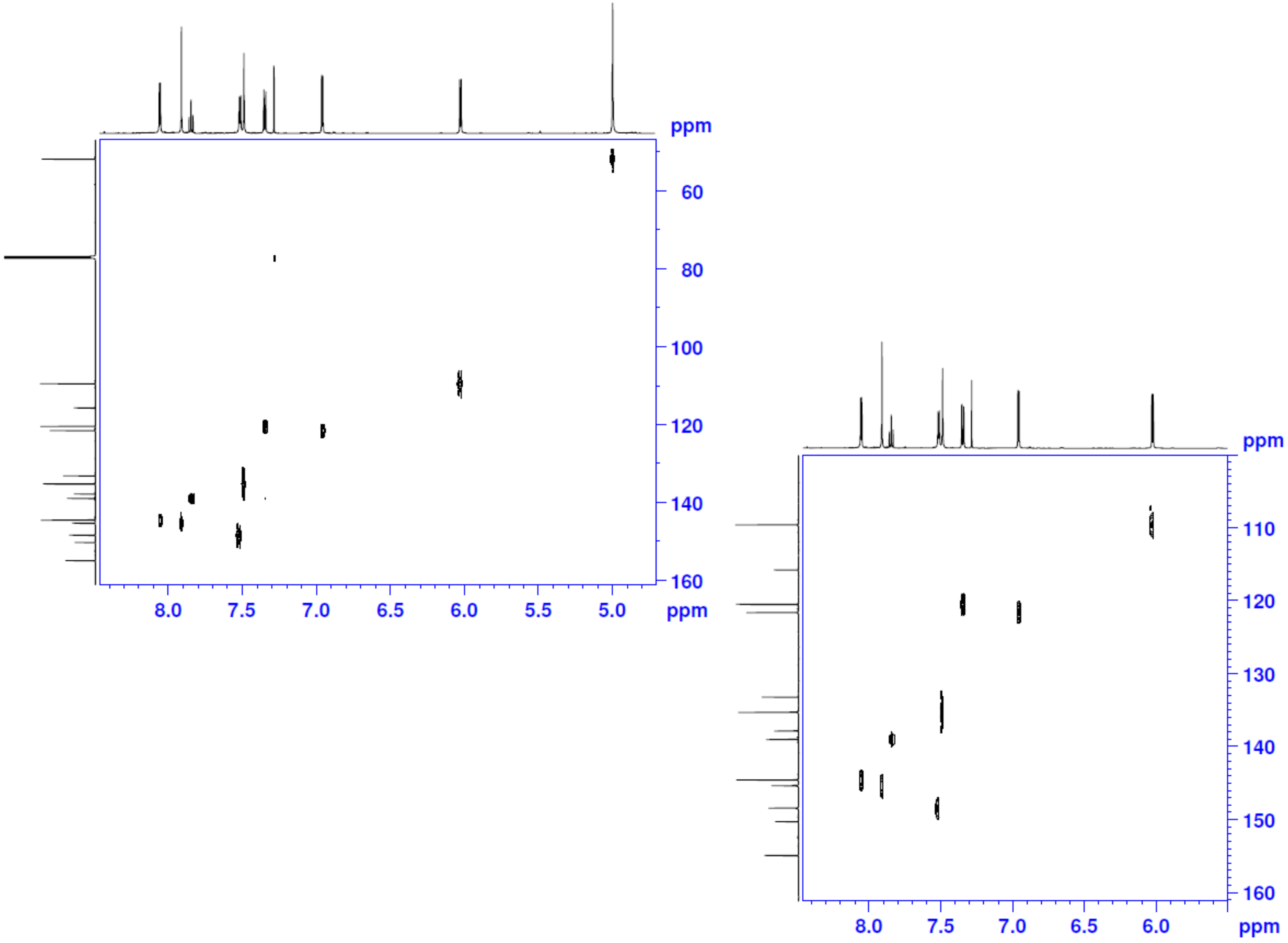

HMBC NMR of 2,6-bis((dipyrido[3,4-b:3',4'-e][1,4]thiazin-10-yl)methyl)pyridine (**3c**)

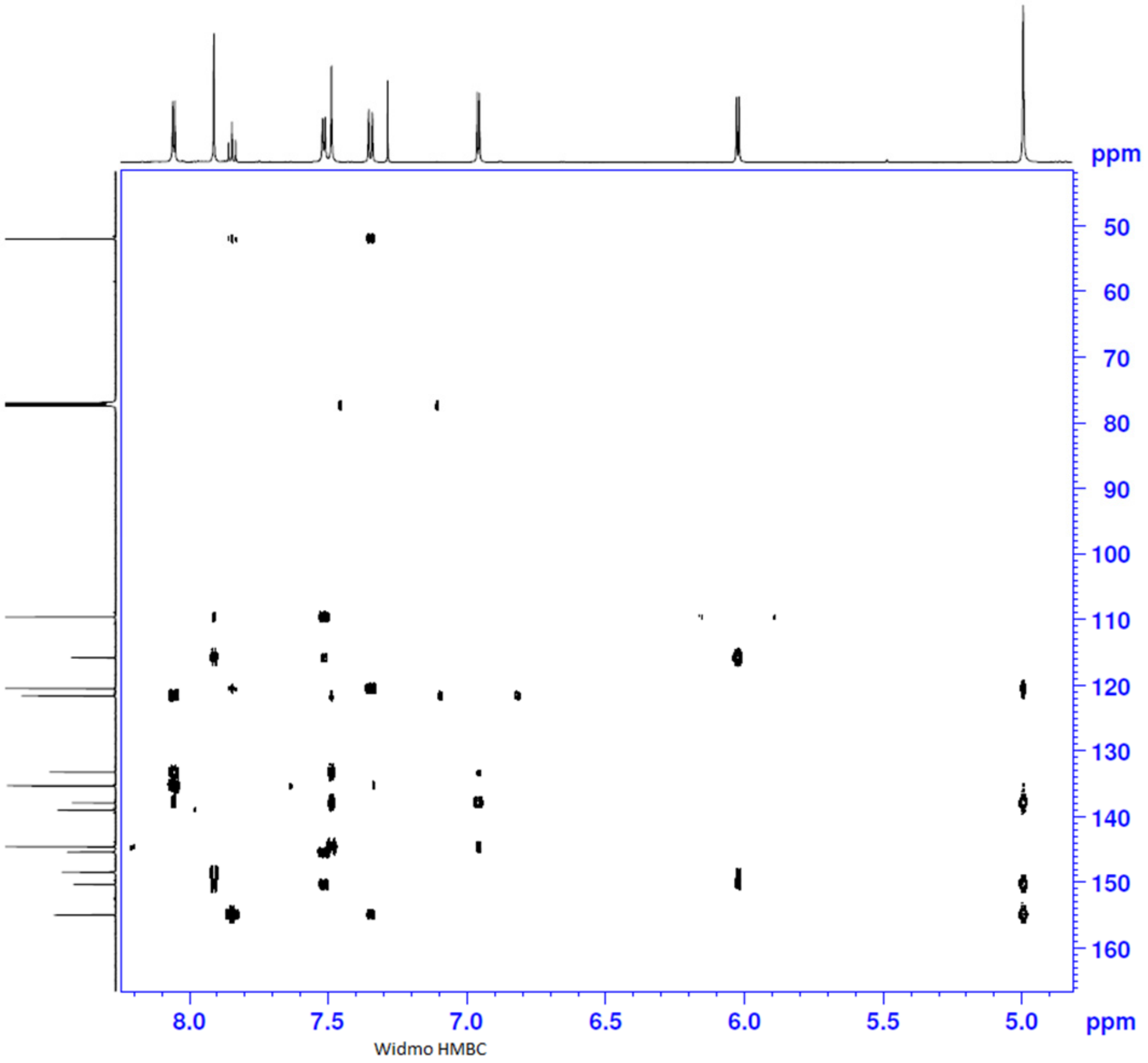

HR MS of 2,6-bis((dipyrido[3,4-b:3',4'-e][1,4]thiazin-10-yl)methyl)pyridine (**3c**)

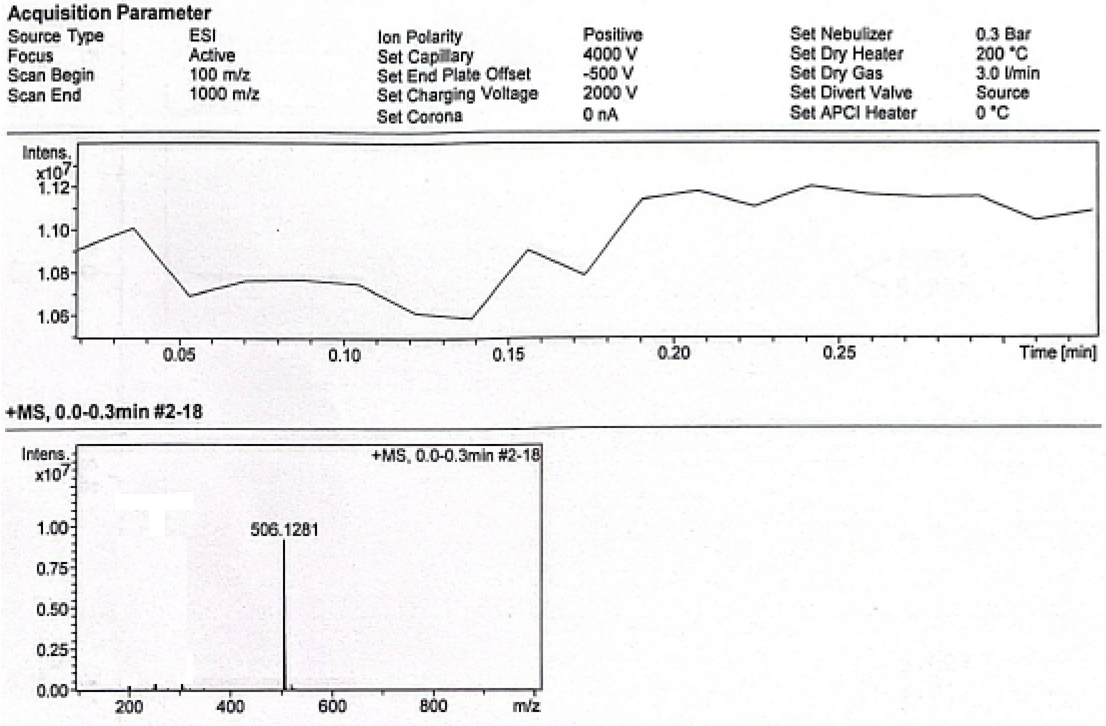

NMR spectra and HR MS of 1,2-bis((dipyrido[2,3-b:4',3'-e][1,4]thiazin-5-yl)methyl)benzene (**4a**)

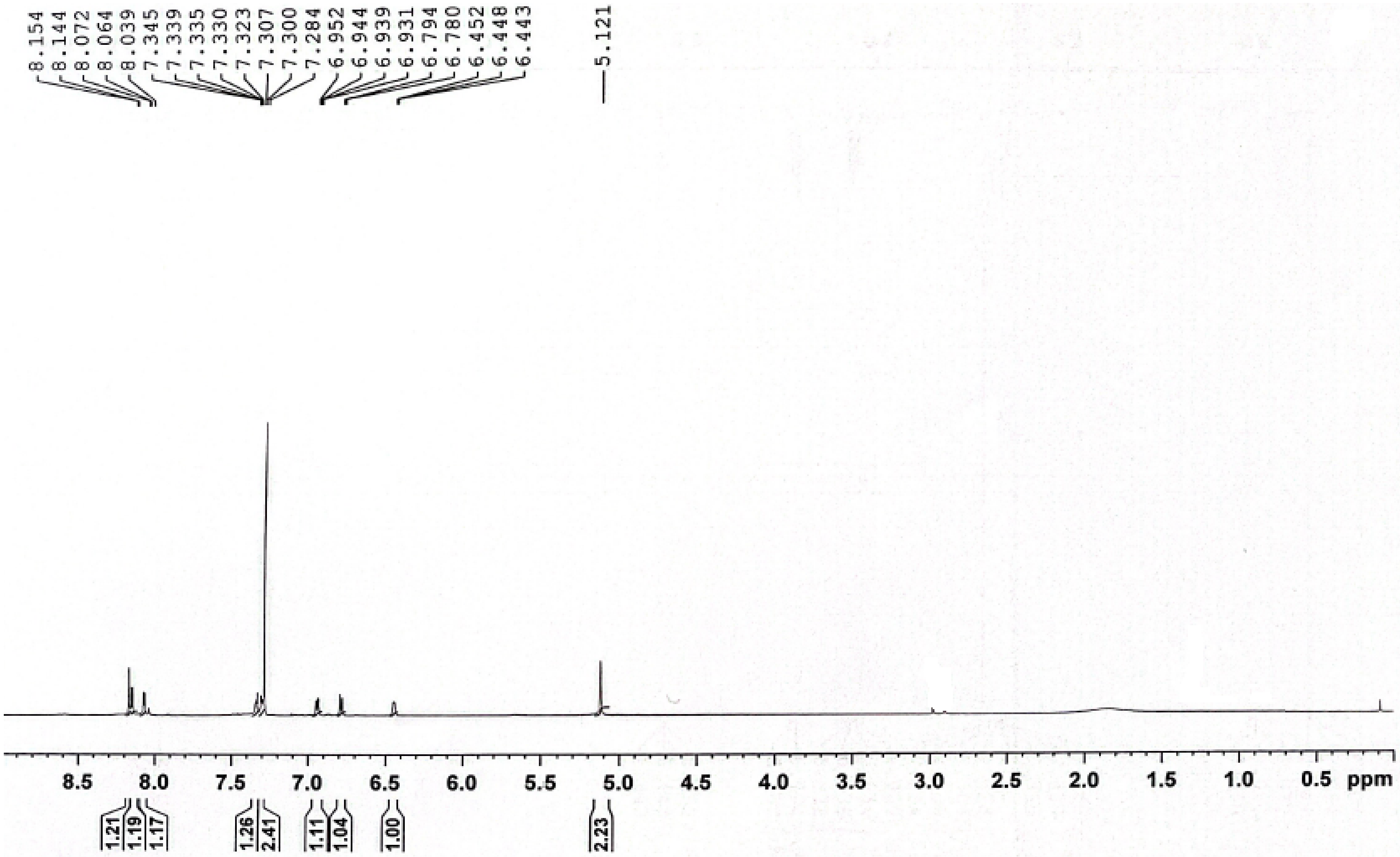
8.154
8.144
8.072
8.064
8.039
7.345
7.339
7.335
7.330
7.323
7.307
7.300
7.284
6.952
6.944
6.939
6.931
6.794
6.780
6.452
6.448
6.443
5.121
8.5
8.0
7.5
7.0
6.5
6.0
5.5
5.0
4.5
4.0
3.5
3.0
2.5
2.0
1.5
1.0
0.5
ppm
1.21
1.19
1.17
1.26
2.41
1.11
1.04
1.00
2.23

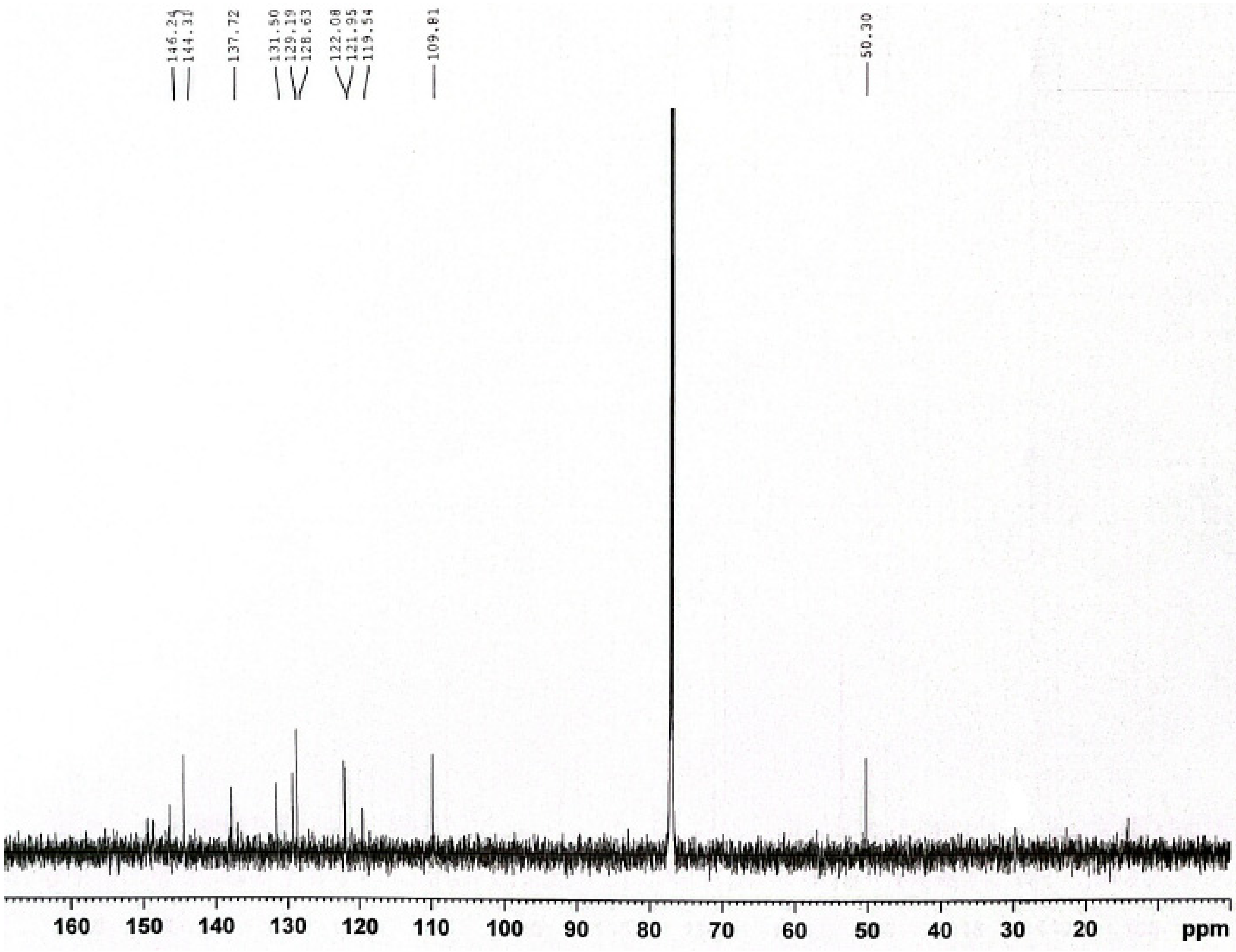
146.24
144.31
137.72
131.50
129.19
128.63
122.08
121.95
119.54
109.81
50.30
160
150
140
130
120
110
100
90
80
70
60
50
40
30
20
ppm

Acquisition Parameter

Source Type	ESI	Ion Polarity	Positive	Set Nebulizer	0.3 Bar
Focus	Active	Set Capillary	3900 V	Set Dry Heater	200 °C
Scan Begin	50 m/z	Set End Plate Offset	-500 V	Set Dry Gas	4.0 l/min
Scan End	1000 m/z	Set Charging Voltage	2000 V	Set Divert Valve	Source
		Set Corona	0 nA	Set APCI Heater	0 °C

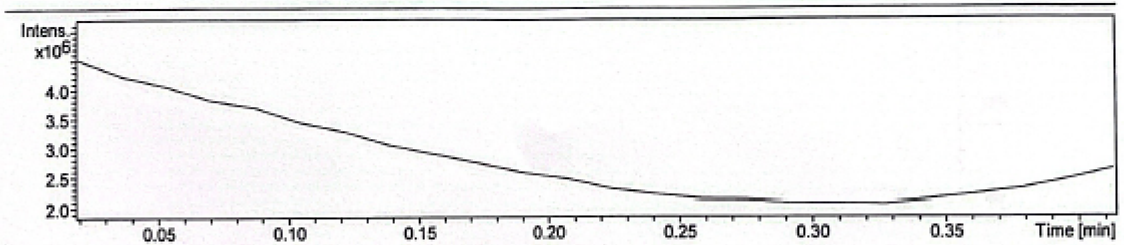

+MS, 0.1-0.4min #4-23

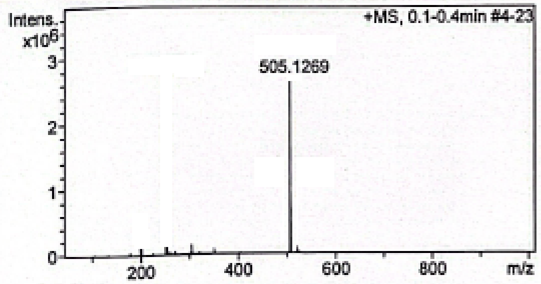

NMR spectra and HR MS of 1,4-bis((dipyrido[2,3-b:2',3'-e][1,4]thiazin-10-yl)methyl)benzene (**4b**)

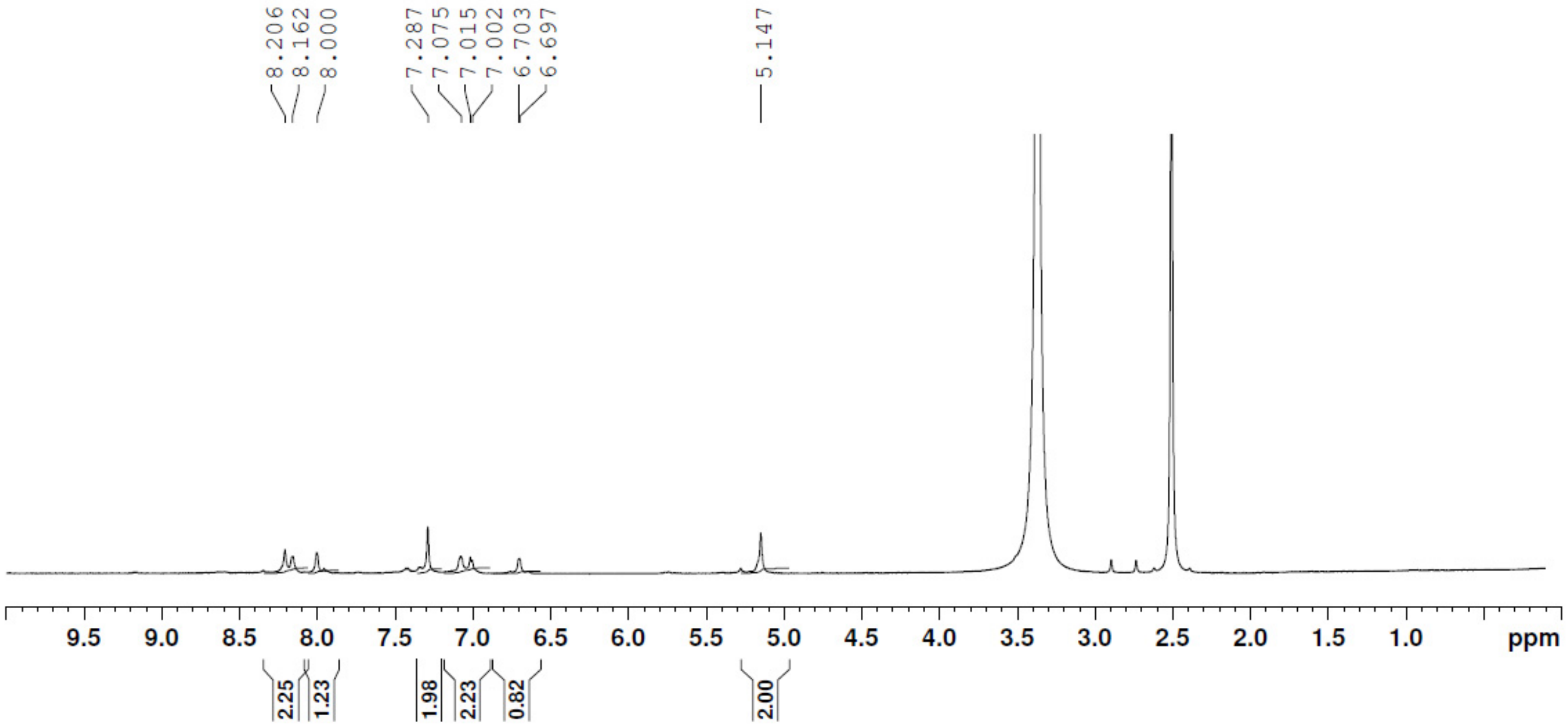

Acquisition Parameter

Source Type	ESI	Ion Polarity	Positive	Set Nebulizer	0.3 Bar
Focus	Active	Set Capillary	3900 V	Set Dry Heater	200 °C
Scan Begin	50 m/z	Set End Plate Offset	-500 V	Set Dry Gas	4.0 l/min
Scan End	1000 m/z	Set Charging Voltage	2000 V	Set Divert Valve	Source
		Set Corona	0 nA	Set APCI Heater	0 °C

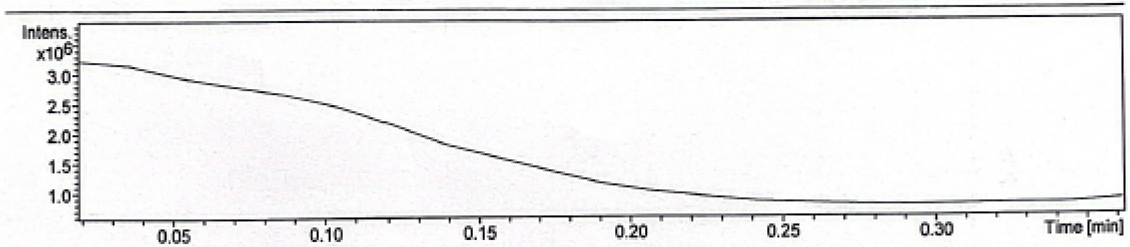

+MS, 0.1-0.3min #4-20

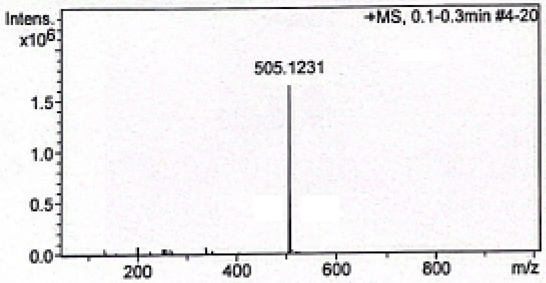

NMR spectra and HR MS of 2,6-bis((dipyrido[2,3-b:4',3'-e][1,4]thiazin-10-yl)methyl)pyridine (**4c**)

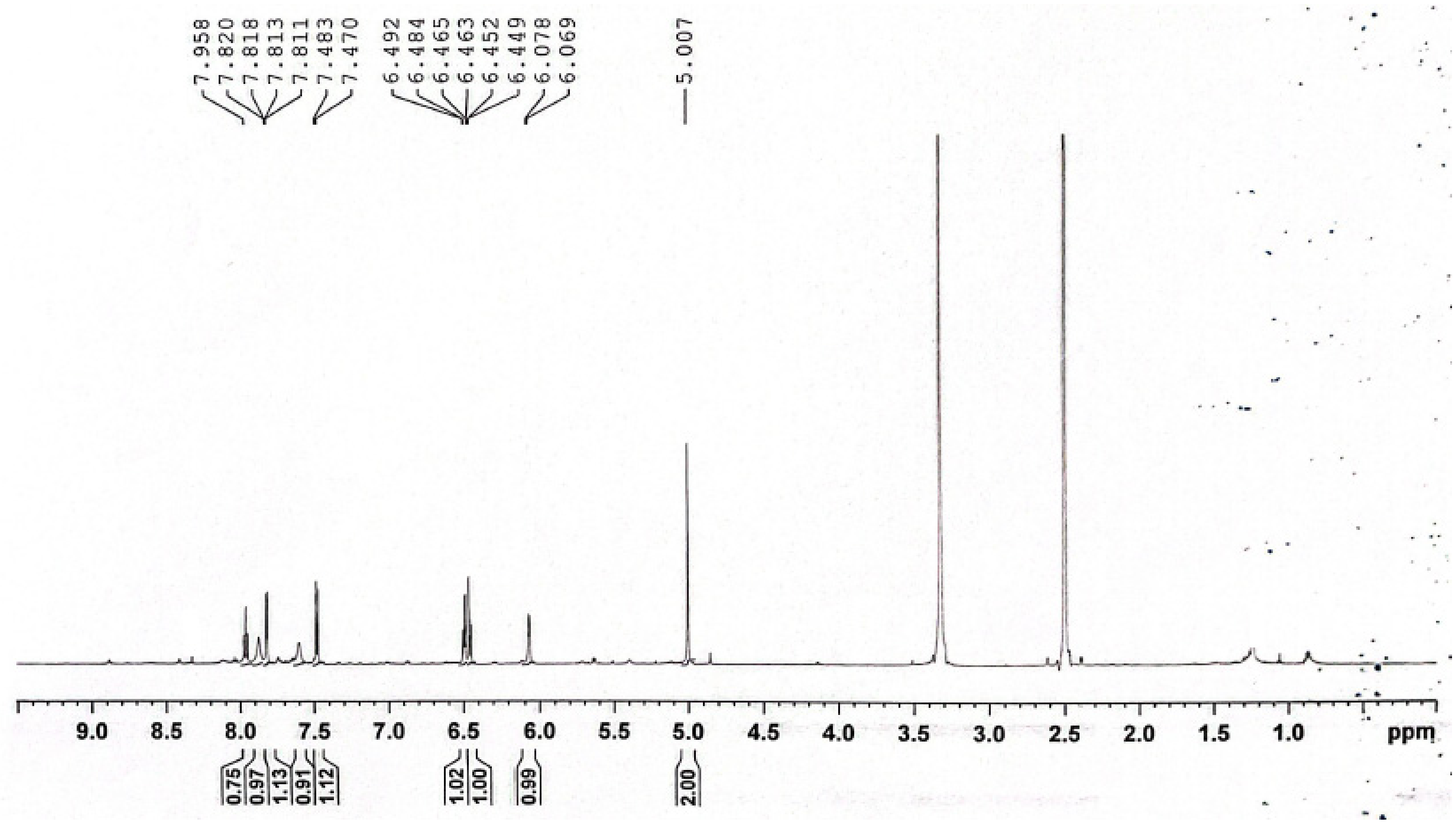

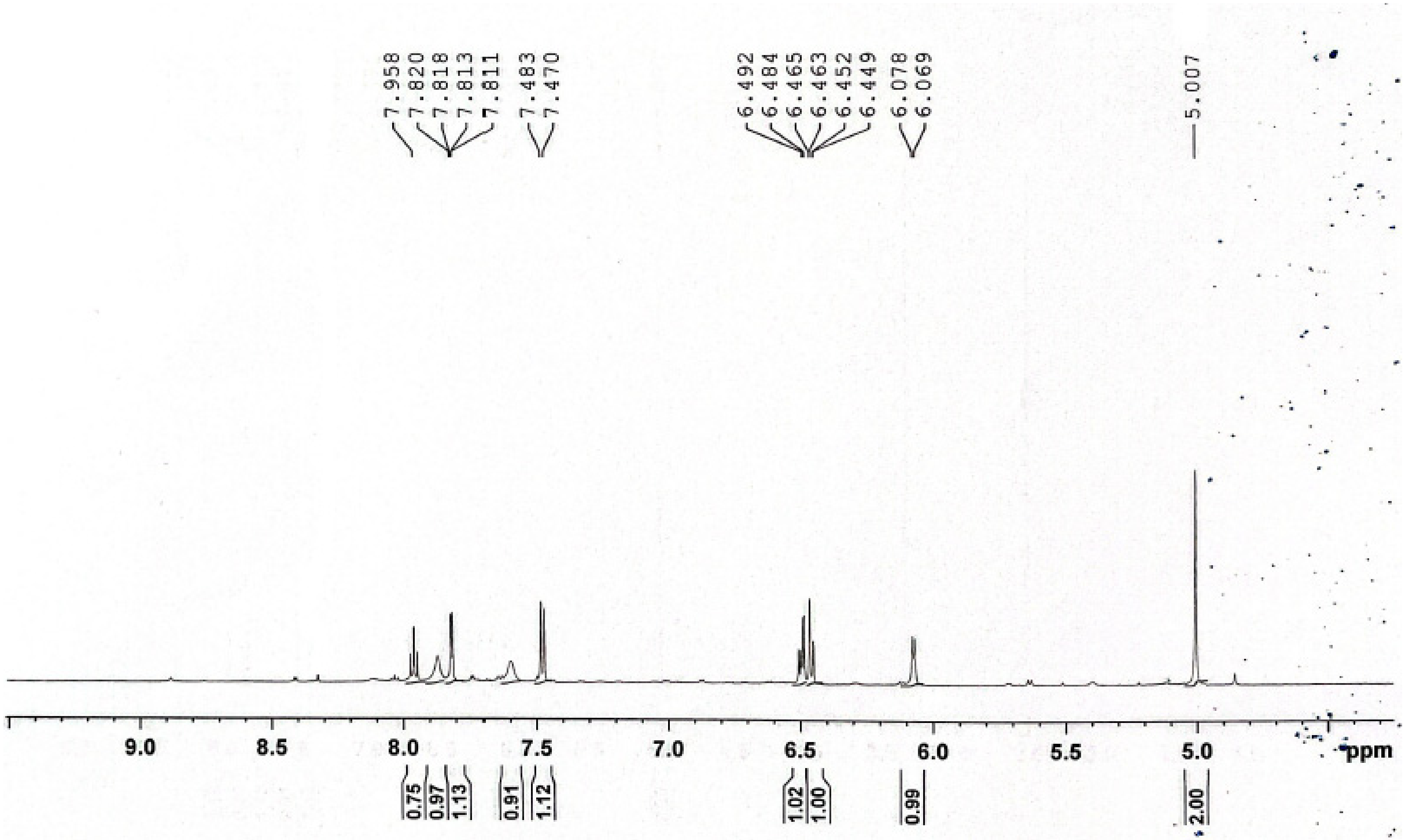
7.958
7.820
7.818
7.813
7.811
7.483
7.470
6.492
6.484
6.465
6.463
6.452
6.449
6.078
6.069
5.007
9.0
8.5
8.0
7.5
7.0
6.5
6.0
5.5
5.0
ppm
0.75
0.97
1.13
0.91
1.12
1.02
1.00
0.99
2.00

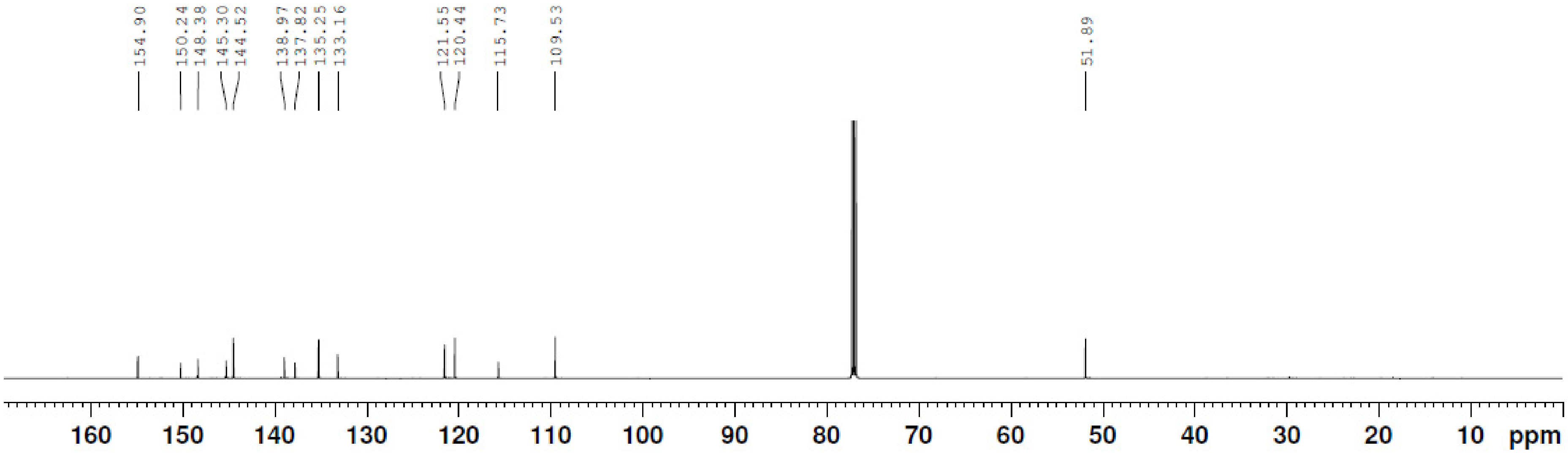
154.90
150.24
148.38
145.30
144.52
138.97
137.82
135.25
133.16
121.55
120.44
115.73
109.53
51.89
160
150
140
130
120
110
100
90
80
70
60
50
40
30
20
10
ppm

Acquisition Parameter

Source Type	ESI	Ion Polarity	Positive	Set Nebulizer	0.3 Bar
Focus	Active	Set Capillary	3900 V	Set Dry Heater	200 °C
Scan Begin	50 m/z	Set End Plate Offset	-500 V	Set Dry Gas	4.0 l/min
Scan End	1000 m/z	Set Charging Voltage	2000 V	Set Divert Valve	Source
		Set Corona	0 nA	Set APCI Heater	0 °C

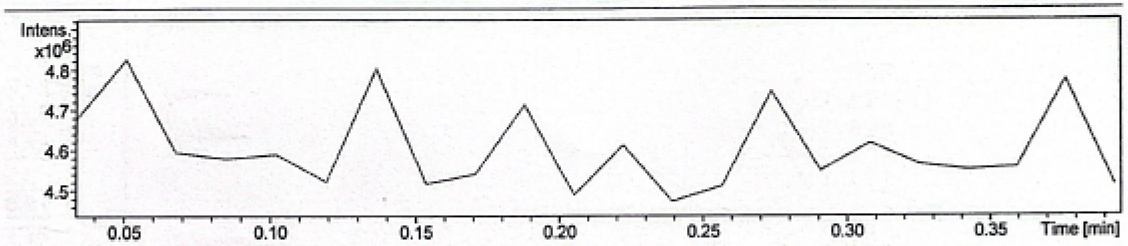

+MS, 0.3-0.4min #16-21

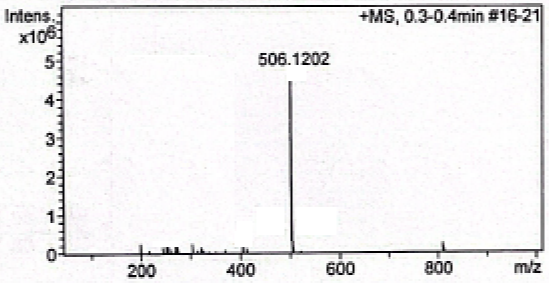

Supplement: Supplementary file 1 [file molecules-28-07662-s001.zip › molecules-2725161-supplementary.pdf]
